# Single Nucleotide Polymorphisms of IL-33 Gene Correlated with Renal Allograft Fibrosis in Kidney Transplant Recipients

**DOI:** 10.1155/2021/8029180

**Published:** 2021-12-13

**Authors:** Xuzhong Liu, Kun Liu, Zeping Gui, Dengyuan Feng, Zijie Wang, Ming Zheng, Shuang Fei, Hao Chen, Li Sun, Zhijian Han, Xiaobing Ju, Hengcheng Zhang, Ruoyun Tan, Min Gu

**Affiliations:** ^1^Department of Urology, The First Affiliated Hospital with Nanjing Medical University, Nanjing 210029, China; ^2^Department of Urology, Huai'an First People's Hospital, Nanjing Medical University, Huai'an, 223300, China; ^3^Department of Urology, The Second Affiliated Hospital with Nanjing Medical University, Nanjing 210011, China; ^4^Transplantation Research Center, Brigham and Women's Hospital, Harvard Medical School, Boston 02115, USA

## Abstract

**Background:**

Nowadays, renal allograft survival is confined by the development of allograft fibrosis. Previous studies have reported interleukin-33 (IL-33) upregulated significantly in patients with chronic renal allograft dysfunction, and it could induce renal tubular epithelial to mesenchymal transition (EMT), which eventually contributed to renal allograft fibrosis. Our study intended to detect the underlying association between single nucleotide polymorphisms (SNPs) of IL-33 gene and renal allograft fibrosis in kidney transplant recipients.

**Methods:**

We collected blood samples from 200 renal transplant recipients for the identification of SNPs and transplanted kidney tissue samples for identifying differentially expressed genes (DEGs). Intersection of SNP-related genes and DEGs was conducted for further analysis. Relationships between these SNPs and renal allograft fibrosis were evaluated by the inheritance models. Immunohistochemical (IHC) staining and western blotting (WB) were used to detect the expression of IL-33 and the markers of EMT in human kidney tissues obtained from control and chronic renal allograft dysfunction (CAD) patients. In vitro, we detected the progressions of EMT-related markers and the levels of MAPK signaling pathway mediators after transfecting IL-33 mutant plasmids in HK2 cells.

**Results:**

Three intersected genes including IL-33 genes were significantly expressed. IL-33 expression was validated in kidney tissues by IHC and WB. Thirty-nine IL-33-related SNPs were identified in targeted sequencing, in which 26 tagger SNPs were found by linkage disequilibrium analysis for further analysis. General linear models indicated sirolimus administration significantly influenced renal allograft fibrosis (*P* < 0.05), adjustment of which was conducted in the following analysis. By multiple inheritance model analyses, SNP rs10975519 of IL-33 gene was found closely related to renal allograft fibrosis (*P* < 0.005). Furthermore, HK2 cells transfected with mutated plasmid of rs10975519 showed stronger mobility and migration ability. Moreover, IL-33 mutant plasmids could promote the IL-33-induced EMT through the sustained activation of p38 MAPK signaling pathway in HK2 cells.

**Conclusion:**

In our study, rs10975519 on the IL-33 gene was found to be statistically associated with the development of renal allograft fibrosis in kidney transplant recipients. This process may be related to the IL-33-induced EMT and sustained activation of p38 MAPK signaling pathway.

## 1. Introduction

Kidney transplantation is the optimal choice of treatments for patients with end-stage renal disease [1]. However, allograft survival is confined by the development of renal graft fibrosis, which is the ultimate common pathway occurring in almost every type of progressive renal diseases and results in tissue structure and microperfusion destruction leading to graft failure [2]. In renal allografts, interstitial fibrosis and tubular atrophy is termed as IF/TA, whose incidence increases with time [3, 4]. Various immune and nonimmune injuries contribute to the lesion of IF/TA [5], and a multifactorial and finely interregulated biological network induces the pathogenic process of renal allograft fibrosis [6]. Generation of fibrosis in the renal allograft is driven by numerous factors including inflammation, infections, immunosuppressive therapies, and genetic factors [7]. In the previous studies, genetic polymorphisms such as caveolin-1 and SHROOM3 promoted the formation of renal fibrosis and increased risks of allograft failure [8, 9], reminding genetic factors are worth of further investigation in the exploration of renal graft fibrosis.

Nowadays, gene mutation has been testified participating in various pathophysiological processes of organs and tissue fibrosis, which provide new strategies into diagnosis and treatment of renal allograft fibrosis [10]. Plenty of studies clarified correlation between single nucleotide polymorphisms (SNPs) of related genes and renal allograft fibrosis. Domanski et al. illustrated rs5498 ICAM1 gene polymorphism was associated with the grade of renal allograft interstitial fibrosis, and presence of G allele indicated more severe prognosis [11]. rs17319721 on SHROOM3 gene could promote TGF-*β*1 signaling and contribute to renal allograft injury and interstitial fibrosis [9]. Furthermore, the CAV1 rs4730751 SNP independently predicted transplanted renal fibrosis and allograft failure, and the late allograft biopsies showed the incidence of renal IF/TA was higher in the AA group (59% vs. 26%) [8].

Interleukin-33 (IL-33) as a member of IL-1 superfamily of cytokines, interacting with its receptors including suppression of tumorigenicity 2 (ST2) and IL-1 receptor accessory protein (IL-1 RACP), activates nuclear factor-*κ*B and mitogen-activated protein kinase (MAPK) signaling pathways and induces T helper 2 cell-associated cytokine production to exert its biological effects [12, 13]. Emerging data indicated that the IL-33/ST2 pathway predominantly contributed to the pathophysiological processes of organ fibrosis including liver, heart, and kidney [14]. IL-33 was constitutively expressed in peritubular capillary endothelial cells in human kidney specimens [15], whose expression was associated with progressive deterioration of renal function [16]. According to our previous study in kidney transplantation, IL-33 was significantly upregulated in patients with chronic allograft dysfunction compared with recipients with stable allograft function and healthy volunteers [17]. Epithelial to mesenchymal transition (EMT) of podocytes, tubular epithelial cells, and circulating fibrocytes constitutes the principal mechanism of renal fibrosis [18]. Our study *in vitro* also manifested IL-33 could induce EMT and facilitate capability of cell motility and migration in HK2 cells [19]. Hence, we speculated that SNPs of IL-33 may have an impact on the occurrence of renal graft fibrosis.

In our study, comparative research of IL-33-related SNPs was performed in kidney transplant recipients with next-generation sequencing (NGS) application to investigate the underlying relationship between IL-33 SNPs and renal allograft fibrosis after kidney transplantation.

## 2. Material and Methods

### 2.1. Ethics Statement

The study was in accordance with the guidelines of the Declaration of Helsinki, and all procedures involving human participants were approved by the ethics committees of the First Affiliated Hospital with Nanjing Medical University (2016-SR-029). Written informed consents were obtained from all the recipients involved.

### 2.2. SNP Detection

#### 2.2.1. Study Design

This was a retrospective case-control study with 200 participants, who underwent kidney transplantation surgery between 1st February 2010 and 1st December 2015, at the First Affiliated Hospital with Nanjing Medical University. The included recipients met the following inclusion criteria: (1) recipients who were more than 18 years or less than 60 years, (2) recipients who either experienced stable serum creatinine levels (<120 *μ*mol/L; fluctuation < 20%) for at least 3 months or were diagnosed with chronic allograft dysfunction (CAD) by laboratory and pathological examinations, and (3) recipients with follow-up for more than 6 months after kidney transplantation. The exclusion criteria included are as follows: (1) recipients who did not meet the inclusion criteria; (2) recipients with severe heart, liver, or lung disease or chronic viral infections; and (3) recipients diagnosed with pregnancy and lactation.

CAD is characterized by progressive proteinuria and hypertension, as well as a gradual increase in serum creatinine level over months, along with aberrant urine output and edema [20]. Recipients with the mentioned clinical manifestation and corresponding graft ultrasonic display were considered with the occurrence of CAD. Renal allograft biopsies were performed in some recipients who signed informed consent of biopsy for further validation. Banff criteria (2017) provided pathological diagnosis standard with the application of molecular biological technique [21]. All enrolled recipients were assigned into the CAD group and the control group.

Demographic data of recipients, such as age, sex, and panel reactive antibodies (PRAs) at the time of kidney transplantation and immunosuppressive protocols, were reviewed from the medical records (Table 1).

#### 2.2.2. Immunosuppressive Protocols

Induction therapy at kidney transplantation was performed using basiliximab or antihuman thymocyte immunoglobulin. All participants received immunosuppressive regimens, including calcineurin inhibitors such as tacrolimus or cyclosporin A, mycophenolate mofetil, and prednisone, with or without sirolimus during the maintenance period. Immunosuppression protocols were elaborated in this previous paper [22]. The dosage of immunosuppressants was adjusted according to the serum creatinine level and drug concentration.

#### 2.2.3. Preparation of Sample and Analysis of Target Sequencing

Sample collection and preparation as well as target sequencing (TS) analysis were thoroughly explained in a related paper [22]. Peripheral blood samples (2 ml) were taken from recipients and then stored at -80°C. DNA extraction from blood samples was completed with the QIAmp DNA mini kit (Qiagen, Hilden, Germany). The genomic DNA (gDNA) was purified and concentrated for quantitative analysis using NanoDrop ND2000 (Thermo, MA, USA). Then, target regions of interest were selected as gDNA hybrids. The gDNA was further fragmented into pieces for quantitative detection using a Bioruptor Interrupt (Diagenode, Belgium) to ascertain the average fragment size was 150–250 bp. The adapter-ligated DNA was amplified using polymerase chain reaction (PCR) for 5 cycles and then analyzed using the Qubit dsDNA HS assay kit (Invitrogen, USA). The captured libraries were hybridized, denatured, and loaded onto an Illumina cBot instrument based on the instructions from manufacturer. Sequencing data derived from available data were analyzed according to the human reference sequence UCSC hg19 assembly (NCBI build 37.2), using Genome Analysis Tool Kit, Picard Software, and dbSNP 132. Moreover, two separate programs, MuTect 1.1.5 and VarScan 2.3.6, were applied for detecting putative somatic variant cells. Eventually, the FASTQ files were generated and further compiled into TS data containing SNPs, which were analyzed and interpreted in our study.

### 2.3. Kidney Transplant Tissue Samples

We collected kidney transplant tissues in 3 recipients who were subjected to transplanted kidney nephrectomy due to CAD operated at the First Affiliated Hospital with Nanjing Medical University between 2016 and 2018. In addition, 3 normal kidney samples were obtained from patients undergoing radical nephrectomy, and each sample was excised at 5 cm away from tumor tissue. The collected samples were stored at -80°C for the preparation of RNA extraction. And all the samples were analyzed separately. The baseline characteristics of the CAD and normal groups are described in Table 2.

### 2.4. Identification and Elucidation of DEGs

The prepared samples were further conducted sequencing using the Illumina HiSeq2500 platform according to the manufacturer's instructions. Differentially expressed genes (DEGs) were screened with ∣log fold − change (FC)  | >1 and *P* value < 0.05 between the CAD and normal groups, using the limma R package (version 3.13). The gene ontology (GO) functional annotation of the identified DEGs was performed on the DAVID 6.8 database (https://david.ncifcrf.gov/). *P* value < 0.05 was considered statistically significant.

### 2.5. Cell Culture and Transfection

HK2 cells were cultured in Dulbecco's modified Eagle's medium (DMEM)/F12 medium (Invitrogen, USA) containing 10% fetal bovine serum s in a humidified atmosphere containing 5% CO2 at 37°C.

The pCMV-IL-33 or pCDNA3.1 plasmids were constructed and synthesized by Jikai company (Shanghai, China). Transfection was performed using Lipofectamine 3000 kit (Invitrogen, USA) according to the manufacturer's protocol. After transfection for 24 h, HK2 cells were treated with 10 ng/ml IL-33 for 0, 2, 4, 8, 12, and 24 h. Total protein and RNA were extracted for western blot assays and quantitative real-time PCR (qRT-PCR), respectively.

### 2.6. Western Blot

Cultured HK2 cells or tissues were lysed with RIPA buffer (Thermo ScientificTM, Chelmsford, MA, USA) containing phosphatases and proteases inhibitor cocktails (Sigma, St Louis, MO, USA) to obtain proteins. Proper proteins were loaded into 10% sodium dodecyl sulfate-polyacrylamide gel electrophoresis and transferred onto polyvinylidene difluoride membrane (Millipore, IPVH00010, Massachusetts, USA). The primary antibodies were listed as follows: anti-GAPDH (1 : 1000; CST, USA), anti-E-cadherin (1 : 1000; CST, USA), anti-a-SMA (1 : 1000; Abcam, USA), anti-IL-33 (1 : 1000; Abcam, USA), anti-fibronectin (1 : 1000; BD Biosciences, USA), anti-p-Erk1/2 (1 : 1000; CST, USA), anti-Erk1/2 (1 : 1000; CST, USA), anti-p-p38 (1 : 1000; CST, USA), anti-p38 (1 : 1000; CST, USA), anti-p-c-Jun (1 : 1000; CST, USA), and anti-c-Jun (1 : 1000; CST, USA). This was followed by incubation with an anti-rabbit or anti-mouse secondary antibody (1 : 5000; Abcam, USA). The relative abundance of proteins was quantitatively analyzed using the NIH image analysis software.

### 2.7. Immunohistochemical Staining Assay

Kidney tissues were fixed with 10% formalin and cut into 3 *μ*m thick paraffin sections, which were deparaffinized in xylene and rehydrated in a graded series of alcohol. Nonspecific epitopes were blocked with 5% normal goat serum for 30 min, followed by incubation with anti-IL-33 (1 : 2000; Abcam, USA) over night at 4°C. Sections were incubated with biotinylated goat anti-mouse/rabbit IgG (5.0 *μ*g/ml; Abcam) for 1 hour. Slides were observed with a light microscope equipped with a digital camera (ECLIPSE 80i; Nikon).

### 2.8. HK2 Cell Mobility Assay

Mobility assay with HK2 cells transfected with pCMV-IL-33(mut) or pCDNA3.1 plasmids was implemented in 6-well culture dishes. After transfection for 24 h, we scratched HK2 cells with pipette tips. Images were taken after scratching cells for 0 h and 24 h, using an inverted microscope (Eclipse TS100; Nikon, Shinagawa, Tokyo, Japan) at a ×100 magnification. Then, quantitative analysis of the migrated cells was measured by the mobility index (mobility index = the number of cells that migrated in the pCDNA3.1 group/the number of cells that migrated in the pCDNA3.1 group or the mutated group). The assay was repeated at least 3 times independently.

### 2.9. HK2 Cell Migration Assay

The migration ability of HK2 cells transfected with the pCMV-IL-33 or pCDNA3.1 plasmids was determined by transwell assay, which was performed in 24-well culture plates. HK2 cells were seeded on the upper chamber of 8 *μ*m pore polycarbonate filters at a density of 5 × 10^4^ cells. After incubating at 37°C for 48 h, the number of cells migrating to the outsider surface was calculated by a phase-contrast microscope (Eclipse TS100; Nikon) at a ×100 magnification. Quantification of the migrated cells was measured by the migration index (migration index = the number of cells that migrated in the pCDNA3.1 group or the mutated group/the number of cells that migrated in the pCDNA3.1 group). The assay was repeated at least 3 times independently.

### 2.10. Quantitative Real-Time PCR Analysis

Total RNA was harvested from HK2 cells with the RNA extraction kits (TIANGEN, Beijing, China). cDNA was synthesized with a PrimeScript™ RT reagent kit (TaKaRa Biotechnology, Shiga, Japan). qRT-PCR was operated on a DNA Engine Opticon 2 System (Bio-Rad laboratories, Hercules, CA, USA). The primers were listed as follows:

IL-33: 5′-GTGACGGTGTTGATGGTAAGAT-3′ (F) and 5′-AGCTCCACAGAGTGTTCCTTG-3′ (R) and actin: 5′-TGACGTGGACATCCGCAAAG-3′ (F) and 5′-CTGGAAGGTGGACAGCGAGG-3′ (R).

### 2.11. Statistical Analysis

The Hardy-Weinberg equilibrium (HWE) analysis was calculated by the RStudio version 4.0.5 software (Boston, MA, USA) implemented by the “SNPassoc” package version 2.0-2, while minor allele frequencies (MAFs) and the linkage disequilibrium (LD) blocks were computed using the Haploview version 4.2 software (Broad Institute, Cambridge, MA, USA). General linear models (GLMs) were applied for figuring out the potential connection between clinical variables and renal allograft fibrosis. Genotype association analysis was evaluated by five inheritance models including dominant model (minor allele homozygotes plus heterozygotes vs. major allele homozygotes), recessive model (minor allele homozygotes vs. heterozygotes plus major homozygotes), codominant model (major allele homozygotes vs. heterozygotes vs. minor allele homozygotes), overdominant model (heterozygotes versus major allele homozygotes plus minor allele homozygotes), and log-additive model, using the RStudio version 4.0.5 software with Package SNPassoc (version 2.0-2). Comparison of genotypic frequencies between the CAD group and the control group was completed by Chi-square test. The data were analyzed by the SPSS version 25.0 software (SPSS Inc., Chicago, IL, USA), and *P* < 0.05 was considered statistically significant.

## 3. Results

### 3.1. Baseline Characteristics of Control and CAD Group Recipients

Two hundred recipients meeting the inclusion criteria were enrolled in this study. The baseline characteristics of included recipients are displayed in Table 1. The average age of those recipients was 44.59 ± 4.09 years, whose PRAs at the time of kidney transplantation were all negative. All the recipients underwent primary kidney transplantation with the mean HLA matching of 1.01 ± 1.21. Twelve percent of recipients were medicated with sirolimus in the maintenance period. No significant difference was observed between the CAD group and the control group (*P* > 0.05).

### 3.2. Tagger SNP Selection

**Figure 1:**
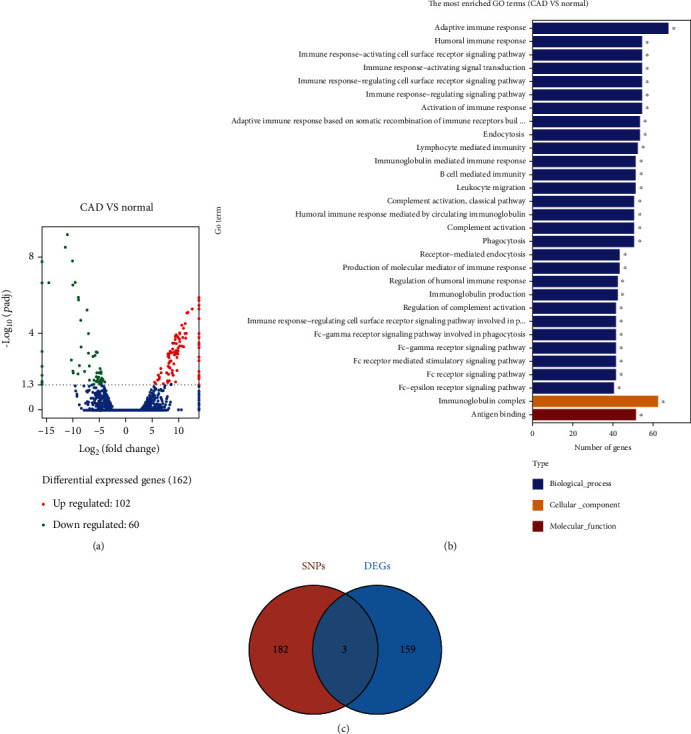
Identification and functional annotation of DEGs and target SNPs between the control and CAD groups. (a) Volcano plots of 162 DEGs were shown between the two groups with 102 upregulated genes and 60 downregulated genes. (b) GO enrichment analysis depicted DEGs were associated with the immune system and inflammatory response. (c) Venn diagram exhibited three intersected genes including IL-33 between DEGs and SNP mutation-related genes. Abbreviations: DEGs: differentially expressed genes; SNP: single nucleotide polymorphism; CAD: chronic renal graft dysfunction; GO: gene ontology.

**Figure 2:**
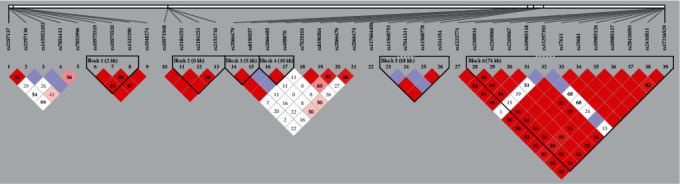
Linkage disequilibrium plot of SNPs in the detected genes. Among these, three IL-33-related SNPs had strong correlations: rs10975519, rs10975520, and rs1332290. Abbreviation: SNPs: single nucleotide polymorphisms.

**Figure 3:**
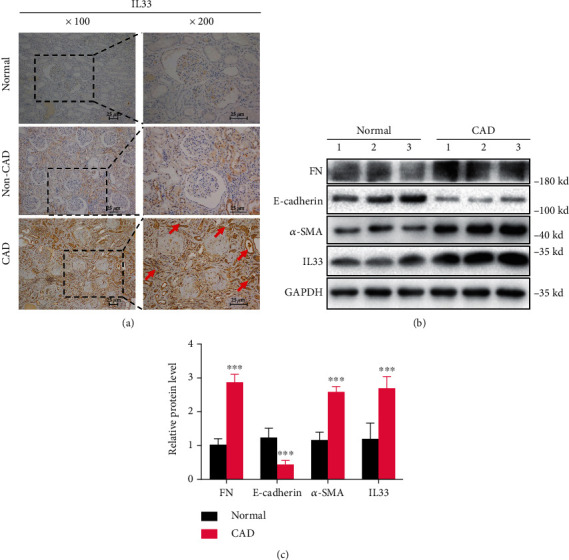
Validation of association between target SNP gene (IL-33) and renal graft fibrosis. (a) IHC staining using anti-IL-33 antibodies demonstrated more deposition of IL-33 in renal tubules (red arrow) was found in graft fibrosis tissues of CAD than normal tissues. Scale bar: 25 *μ*m. (b) Representative WB results showed IL-33 expression as well as EMT-related markers including FN and *α*-SMA in the CAD group exceeded those in the normal group, while E-cadherin expression was higher in the normal group than the CAD group. (c) Semiquantitative analysis results of relative protein abundances of FN, E-cadherin, *α*-SMA, and IL-33 were shown in each group. Values represented the mean ± SD (*n* = 3; ^∗∗∗^*P* < 0.001). Abbreviations: SNP: single nucleotide polymorphism; IHC: immunohistochemistry; CAD: chronic renal graft dysfunction; WB: western blot; EMT: epithelial-mesenchymal transition.

We identified SNPs of 185 genes for further analysis by using HWE analysis and filter criteria of MAF > 0.05 (Supplementary Table 1 and 2). As shown in Figure 1(a), differential expressed genes (DEGs) between control and CAD groups (*n* = 3) were evaluated by RNA sequencing. Red represents an upregulated gene, and green represents a downregulated gene. By comparing the results of sequencing between the two groups, 3,255 transcripts and 1,406 genes were found differentially expressed in the two groups, of which 162 DEGs were screened out, while 102 genes were upregulated, and 60 genes were downregulated, and the absolute value of log2 fold was >1 (*P* < 0.05). GO enrichment analysis suggested that these DEGs were related to the immune system and inflammatory response, such as immunoglobulin complex (GO:0019814), humoral immune response (GO:0006959), adaptive immune response (GO:0002250), leukocyte migration (GO:0050900), and endocytosis (GO:0006897) (Figure 1(b)). For this reason, three genes were selected by taking the intersection of the 162 DEGs set and 185 genes which were SNP mutated (Figure 1(c)). Furthermore, LD analysis was conducted among the SNPs to explore the tagger SNPs (Figure 2). Among the selected three genes, only rs10975519, rs10975520, and rs1332290 of IL-33 gene are located at the first linkage disequilibrium block. Hence, SNPs from IL-33 gene were used as representative loci for the subsequent statistical analysis. The HWE and MAF results of different SNP multilocus on IL-33 gene are shown in Supplemental Table 2. Additionally, we performed IHC and WB verification of the DEG and tagger SNP gene—IL-33 (Figure 3).

### 3.3. Association Analysis of IL-33-Associated SNPs with Renal Allograft Fibrosis

GLMs were implemented for detecting the potential impact of confounding clinical variables on renal allograft fibrosis.  Table 3 shows that recipients with the administration of sirolimus significantly influenced renal allograft fibrosis (*P* < 0.05), while other confounding factors, including occurrence of acute rejection, sex, age, weight, protocol of immunosuppressive drugs, duration after kidney transplantation, and occurrence of delayed graft function, did not (*P* > 0.05).

Hence, applying with Bonferroni correction (adjusted *P* value = 0.005), inheritance model analysis with the adjustment of sirolimus administration was performed in this study. SNP rs10975519 of IL-33 gene was found closely related to renal allograft fibrosis (Table 4: codominant model: OR = 6.05, 95%CI = 2.25–16.27, and *P* < 0.001; dominant model: OR = 2.76, 95%CI = 1.39–5.49, and *P* = 0.003; recessive model: OR = 3.81, 95%CI = 1.60–9.07, and *P* = 0.002; overdominant model: OR = 1.23, 95%CI = 0.66–2.28, and *P* = 0.512; log-additive model: OR = 2.41, 95%CI = 1.49–3.90, and *P* < 0.001), implying that SNP rs10975519 of IL-33 gene may function as a prediction of renal allograft fibrosis, while other tagger SNPs showed no significant association (*P* > 0.005; Supplementary Table 3).

### 3.4. IL-33 rs10975519 Mutant Enhances IL-33-Induced EMT Related to Promote Sustained Activation of p38 MAPK Pathway

**Figure 4:**
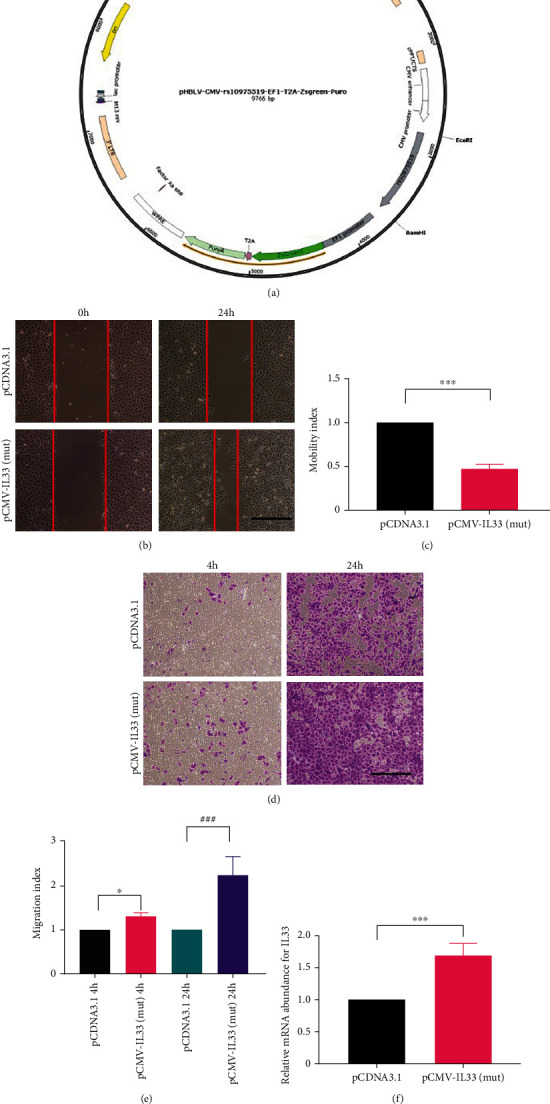
rs10975519 mutation on IL-33 gene promoted the mobility and migration of HK2 cells. (a) Wild-type (pCDNA3.1) and mutated (pCMV-IL-33) plasmids of rs10975519 were constructed for further verification. (b) HK2 cells transfected with pCDNA3.1 or pCMV-IL-33(mut) plasmids were wounded by a pipette (c). Quantitation of the migrated cells was measured by the mobility index, which was determined by the formula “the number of cells that migrated in the pCDNA3.1 group/the number of cells that migrated in the pCDNA3.1 group or the mutated group.” (d) The HK2 cells transfected with the pCMV-IL-33 or pCDNA3.1 plasmids were seeded in the top chamber for 4 h and 24 h. (e) HK2 cells migrating through the membrane were quantified by the migration index, which is calculated by the formula “the number of cells that migrated in the pCDNA3.1 group or the mutated group/the number of cells that migrated in the pCDNA3.1 group.” (f) RT-PCR results indicated higher expression of IL-33 mRNA was seen in the mutated group comparing with the pCDNA3.1 group. Abbreviation: RT-qPCR: real-time PCR.

To identify the biological function of rs10975519 mutation on the IL-33 gene, we transfected the wild-type and mutated plasmids of rs10975519 into HK2 cells, respectively (Figure 4(a)). Wound healing test and transwell assays were performed on HK2 cells to evaluate the acquisition of migratory capacity following treatment with pCMV-IL-33(mut). As shown in Figures 4(b)–4(f), cells transfected with mutated plasmid of rs10975519 showed stronger mobility and migration ability compared to the pCDNA3.1 group. In addition, IL-33 mRNA was upregulated in mutated plasmid transfected cells (Figure 4(f)).

**Figure 5:**
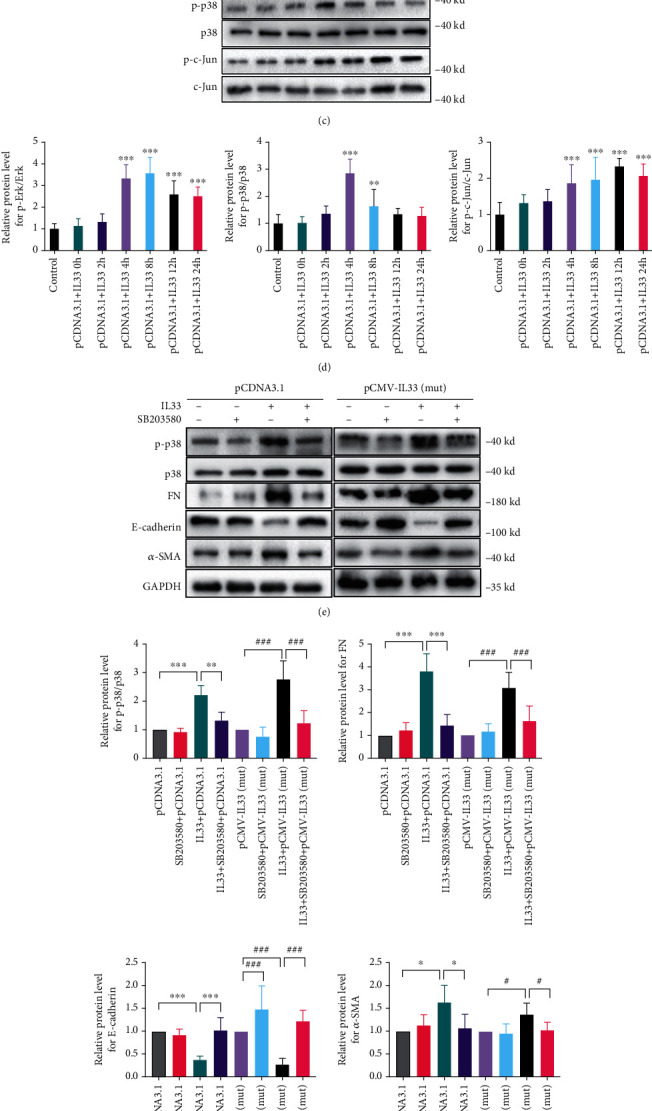
rs10975519 mutation on IL-33 gene contributed to EMT in HK2 cells via activating p38 MAPK signaling pathway. (a) Representative WB results showed rs10975519 mutation of IL-33 gene promoted the expression of EMT-related markers including FN, E-cadherin, and *α*-SMA. (b) Semiquantitative analysis results of relative protein abundances of FN, E-cadherin, *α*-SMA, and IL-33 were shown in each group. Values represented the mean ± SD (*n* = 3; ^∗^*P* < 0.05 and ^∗∗∗^*P* < 0.001 compared with the pCDNA3.1 group). (c) Representative WB results showed that activation in Erk1/2, p38, and the c-Jun MAPK signaling pathways in HK2 cells transfected with pCDNA3.1 and IL-33. (d) Semiquantitative analysis results of relative protein abundances of p-Erk1/2, Erk1/2, p-p38, p38, p-c-Jun MAPK, and the c-Jun MAPK were shown in each group. Values represented the mean ± SD (*n* = 3; ^∗∗^*P* < 0.01 and ^∗∗∗^*P* < 0.001 compared with the control group). (e) Representative WB results showed that activation of p38 MAPK pathway was involved in IL-33-induced EMT in HK2 cells with pCDNA3.1 transfection. (f) Semiquantitative analysis results of relative protein abundances of p-p38, p38, FN, E-cadherin, and *α*-SMA were shown in each group. Values represented the mean ± SD (*n* = 3; ^∗^*P* < 0.05, ^∗∗^*P* < 0.01, and ^∗∗∗^*P* < 0.001 compared with the pCDNA3.1 group; ^#^*P* < 0.05 and ^###^*P* < 0.001 compared with the pCMV-IL-33(mut) group). (g) Representative WB results showed that phosphorylated p38 MAPK level increases continually in rs10975519 mutant comparing with the pCDNA3.1 group, suggesting rs10975519 mutant of IL-33 gene could continuously activate p38 MAPK pathway. (h) Semiquantitative analysis results of relative protein abundances of p-p38 and p38 were shown in each group. Values represented the mean ± SD (*n* = 3; ^∗∗∗^*P* < 0.001 compared with the pCDNA3.1+IL-33 group; ^###^*P* < 0.001 compared with the pCMV-IL-33(mut)+IL-33 group). Abbreviations: EMT: epithelial-mesenchymal transition; MAPK: mitogen-activated protein kinase; FN: fibronectin; *α*-SMA: alpha smooth muscle actin; WB: western blot.

Combined with our previous findings that IL-33 could promote EMT of HK2 cells through activity of p38 MAPK pathway [19], therefore, we first examined the progression of EMT process in mutation of the IL-33 rs10975519 group and neither the mutation group with or without IL-33 cytokines, respectively. WB assay results demonstrated that rs10975519 mutation of IL-33 gene further facilitated the IL-33-induced EMT that EMT marker expressions were significantly higher compared with that in no mutation groups treated with IL-33 alone (Figures 5(a) and 5(b)). We have proved the p38 MAPK signaling pathways were involved in IL-33-induced EMT in blank HK2 cells in our previous study. In this research, we observed the activation in Erk1/2, p38, and the c-Jun MAPK signaling pathways in HK2 cells transfected with pCDNA3.1 and found the three pathways were potently activated in a time-dependent manner by IL-33 (Figures 5(c) and 5(d)). In order to validate p38 MAPK pathway involved in IL-33-induced EMT in HK2 cells with pCDNA3.1 transfection, SB203580 (p38 MAPK inhibitor) was used. After pretreating the HK2 cells with SB203580 for 1 h, the expressions of phosphorylated p38 MAPK, FN, and *α*-SMA were significantly reduced following IL-33 exposure. Conversely, the expression of E-cadherin was significantly increased (Figures 5(e) and 5(f)). In order to gain insights into the mechanism involved in the role of rs10975519 mutant on IL-33-induced EMT, we detected phosphorylated p38 MAPK level for different time. We found that the expression of phosphorylated p38 MAPK peaked at 12 h and then dropped following 24 h with the treatment of IL-33. However, rs10975519 mutant resulted in sustained activation of p38 MAPK, and phosphorylated p38 MAPK level still maintained growth at 24 h after IL-33 treatment (Figures 5(g) and 5(h)).

## 4. Discussion

In our study, we examined the association between SNPs in IL-33 gene and allograft fibrosis in kidney transplantation recipients. A statistically significant correlation was observed between the IL-33 rs10975519 SNP and renal allograft fibrosis. *In vitro* experiments in HK2 cells further validated the results *in vivo*. Previous studies confirmed IL-33 played a crucial part in immune regulation of various diseases including autoimmune diseases, allergic diseases, and organ transplantation [23–25]. Similarity to our results, rs10975519 SNP of the IL-33 gene was associated with the development of ankylosing spondylitis (AS), and the TTCG haplotype formed by s10118795, rs1929992, rs10975519, and rs1048274 indicated a decreased risk of AS [26]. Falahi et al. reported that the TT genotype of rs7044343 C>T on IL-33 gene probably served as a protective factor against allergic rhinitis [27]. Meanwhile, Savenije et al. demonstrated that polymorphisms of IL-33 pathway influenced the development of wheeze and subsequent asthma in early childhood [28].

p38 MAPK, a member of the MAPK family, could mediate the signals which correlated with the generation of renal fibrosis. The impact of p38 MAPK pathway on kidney diseases has been deeply explored in various models. Lv et al. demonstrated that inhibition of p38 MAPK with p38 siRNA could reverse the high glucose-induced EMT in kidney tubular epithelial cells [29]. Analogously, antagonizing the MAPK pathway with pirfenidone attenuated EMT and renal fibrosis in the unilateral ureteral obstruction models [30]. Recently, apoptosis signal-regulating kinase 1 (ASK1), recognized as an upstream regulator for the activation of p38 MAPK, could result in fibrosis and dysfunction in models of kidney disease [31]. Suppression of ASK1 reduced p38 MAPK activation, giving rise to blocking the progression of nephropathy [32]. Our previous study in HK2 cells confirmed the p38 MAPK signaling pathway participated in the pathogenesis of EMT [19]. Consistent with that, by transfecting HK2 cells with wild-type and mutated plasmids of rs10975519 on IL-33, our study further verified p38 MAPK pathway continuously activated via IL-33 contributed to the development of graft fibrosis after transplantation.

Except close relationship between IL-33 and renal allograft fibrosis were investigated in this study, IL-33 was also recognized as a marker of ischemia reperfusion injury (IRI), which contributed to allograft damage in kidney transplantation [33]. IL-33 was expressed in the space around the renal tubules and glomeruli, primarily by microvascular endothelial cells, where it was immediately released during IRI in mice [33]. Meanwhile, Thierry et al. identified that invariant natural killer T cells (iNKTs) were potential targets of IL-33 in IRI [34]. Ferhat et al. further clarified that IL-33 promoted iNKT cell recruitment and subsequent cytokine production including IFN-*γ* and IL-17A, resulting in neutrophil infiltration and activation at the injury site, eventually leading to IRI in transplanted kidney [33, 35]. In renal IRI models, the iNKT cell/IL-33 axis initiated inflammation, while early blockage of IL-33 along with agonist A2AR treatment which attenuated IFN-*γ*-expressing iNKT cell activation, would counteract IRI [36]. Furthermore, Liang et al. reported IL-33 could regulate myeloid fibroblast accumulation, inflammatory cell infiltration, and cytokine and chemokine expression, playing an important part in the pathogenesis of IRI-induced renal fibrosis [37]. Hence, combined with the present results, IL-33 could affect the formation of renal allograft fibrosis through a variety of ways.

According to the previous study, IL-33 is predominantly expressed and stored in the nuclei of epithelial cells and endothelial cells [38]. Nevertheless, the ST2 receptor, which comprises major transcription variants including the full-length transmembrane form (ST2L) and the soluble form (sST2), is primarily expressed by immune cells involved in innate immunity and adaptive immunity [39, 40]. Our study showed genetic factors of IL-33 could promote the development of renal allograft fibrosis. In addition to these, IL-33/ST2 axis has been found close related to graft-versus-host disease (GVHD) [41]. Vander Lugt et al. elucidated that sST2 was a biomarker for the occurrence of GVHD and correlated with relapse and mortality [42]. And blocking the IL-33/ST2 axis with the application of sST2-Fc reduced GVHD development and decreased relevant mortality in the gastrointestinal tract [41]. Meanwhile, ST2 blockage with the administration of a monoclonal antibody restrained the development of GVHD in several mouse models [43]. Yang et al. also reported restriction of the potency of ST2+ regulatory T cells could ameliorate intestinal GVHD [44]. Together with our finding, these reminded us that IL-33 could affect many aspects of transplantation and would be a promising therapeutic target for future treatment.

In conclusion, in our case-control study, 39 IL-33-related SNPs were identified using NGS technology. rs10975519 on the IL-33 gene was found to be statistically associated with the development of graft fibrosis in renal transplantation. For further verifying our results, we conducted *in vitro* experiments to validate our findings and found IL-33 rs10975519 mutant could enhance IL-33-induced EMT. This effect may be related to promote sustained activity of p38 MAPK pathway. Given the known trigger function of IL-33 in HK2 cells, these findings suggest a possible link between SNPs in IL-33 and CAD following kidney transplantation. However, a few limitations still existed in this study. Relatively small sample size might influence the accuracy of the results and conclusion. Confirmation of this mutation was only conducted in HK2 cells *in vitro*, and it would be better to further validate its effect on allograft fibrosis in animal models. Nonetheless, our transcriptomic study provides insight into the etiopathogenesis by which SNP variants of inflammatory cytokine genes may modulate renal allograft interstitial fibrosis.

## Figures and Tables

**Table 1:** Baseline characteristics of included recipients in our study.

Characteristics	All recipients (*n* = 200)	Graft fibrosis group (*n* = 69)	Control group (*n* = 131)	*P* value
Age, years, mean ± SD	40.54 ± 2.03	40.67 ± 2.96	41.29 ± 1.92	NS
Sex (male/female)	119/81	42/27	82/49	NS
PRA at the time of transplantation (%)	0.00	0.00	0.00	NS
Primary/secondary renal transplant	200/0	69/0	131/0	NS
HLA matching	1.01 ± 1.21	1.05 ± 1.26	0.98 ± 1.17	NS
Type of donor	200	69	131	NS
Living-related	24	6	18	
DCD	176	63	113	

Abbreviations: SD: standard deviation; PRA: panel reactive antibody; HLA: human leukocyte antigen; DCD: donation after cardiac death.

**Table 2:** Baseline characteristics of the CAD and normal groups.

Clinical variables	CAD group (*n* = 3)	Normal group (*n* = 3)	*P* value
Age, years, mean ± SD	41.33 ± 4.16	38.00 ± 6.08	NS
Sex, male/female	2/1	2/1	NS
PRA at the time of transplantation (%)	0.00	/	
Primary/secondary renal transplant	3/0	/	
HLA matching, mean ± SD	1.0 ± 1.0	/	
Type of donor, *n*			
Living-related	0	/	
DCD	3	/	

Abbreviations: SD: standard deviation; PRA: panel reactive antibody; HLA: human leukocyte antigen; DCD: donation after cardiac death.

**Table 3:** Influence of confounding factors on the outcomes of graft fibrosis by general linear model.

Confounding factors	OR (95% CI)	*Z* value	*P* value
Age	0.02 (-0.01, 0.06)	1.41	0.16
Sex	-0.12 (-0.89, 0.66)	-0.30	0.76
Weight	-0.01 (-0.05, 0.04)	-0.24	0.81
ISD	-0.29 (-0.96, 0.38)	-0.85	0.39
Duration after renal transplantation	0.00 (0.00, 0.00)	1.29	0.20
Sirolimus administration	-1.08 (-1.85, -0.36)	-2.86	<0.01
History of acute rejection	0.37 (-0.34, 1.08)	1.02	0.31
History of DGF	0.11 (-0.94, 1.11)	0.21	0.83

Abbreviations: ISD: immunosuppressive drugs; DGF: delayed graft function; OR: odds ratio; CI: confidence interval.

**Table 4:** Multiple inheritance analysis of rs10975519 with the adjustment of sirolimus administration.

rs10975519	OR (95% CI)	*P* value
Codominant model	6.05 (2.25, 16.27)	<0.001
Dominant model	2.76 (1.39, 5.49)	0.003
Recessive model	3.81 (1.60, 9.07)	0.002
Overdominant model	1.23 (0.66, 2.28)	0.512
Log-additive model	2.41 (1.49, 3.90)	<0.001

Abbreviations: OR: odds ratio; CI: confidence interval.

## Data Availability

The data used to support the findings of this study are available from the corresponding author upon request.

## Abbreviations

IL-33: Interleukin-33
SNP: Single nucleotide polymorphism
DEGs: Differentially expressed genes
IHC: Immunohistochemical staining
WB: Western blotting
EMT: Epithelial to mesenchymal transition
CAD: Chronic renal allograft dysfunction
GLM: General linear model
IF/TA: Interstitial fibrosis and tubular atrophy
ST2: Suppression of tumorigenicity 2
NGS: Next-generation sequencing
PRAs: Panel reactive antibodies
TS: Target sequencing
gDNA: Genomic DNA
GO: Gene ontology
qRT-PCR: Quantitative real-time PCR
HWE: Hardy-Weinberg equilibrium
MAF: Minor allele frequency
LD: Linkage disequilibrium.

## Supplementary Materials

Supplementary 1 Supplementary Table 1: detailed information of SNPs found in our study.

Supplementary 2 Supplementary Table 2: results of HWE analysis and MAF calculation for included SNPs in our study.

Supplementary 3 Supplementary Table 3: statistical results of five inheritance models in non-significant tagger SNPs.
