# Supplementary material for: Single Nucleotide Polymorphisms of IL-33 Gene Correlated with Renal Allograft Fibrosis in Kidney Transplant Recipients
Source: J Immunol Res. 2021 Dec 13;2021:8029180. doi: 10.1155/2021/8029180 (PMC8689233; doi:10.1155/2021/8029180)
Supplement: Supplementary 1 — Supplementary Table 1: detailed information of SNPs found in our study. [file 8029180.f1.docx]

Supplementary Table 1: Detailed information of SNPs found in our study.

| Chromosome | Location | Reference allele | Alternation allele | Gene name | Function | Gene detail | SNPs |
| --- | --- | --- | --- | --- | --- | --- | --- |
| chr1 | 230845794 | A | G | AGT | exonic | . | rs699 |
| chr1 | 230846679 | T | C | AGT | intronic | . | rs11122576 |
| chr1 | 230840197 | C | A | AGT | intronic | . | rs1926722 |
| chr1 | 230840096 | T | C | AGT | intronic | . | rs1926723 |
| chr1 | 230841559 | G | A | AGT | intronic | . | rs3827749 |
| chr1 | 230840269 | A | G | AGT | intronic | . | rs11122575 |
| chr1 | 230845571 | G | A | AGT | intronic | . | rs28730748 |
| chr1 | 230845977 | G | A | AGT | exonic | . | rs4762 |
| chr1 | 114401231 | A | G | AP4B1-AS1 | ncRNA_intronic | . | rs1217418 |
| chr1 | 114401904 | T | G | AP4B1-AS1 | ncRNA_intronic | . | rs1217419 |
| chr1 | 114377093 | A | G | AP4B1-AS1 | ncRNA_intronic | . | rs1599971 |
| chr1 | 114398821 | G | C | AP4B1-AS1 | ncRNA_intronic | . | rs35173468 |
| chr1 | 114414108 | C | T | AP4B1-AS1 | ncRNA_intronic | . | rs3789612 |
| chr1 | 114372528 | T | G | AP4B1-AS1 | ncRNA_intronic | . | rs3761935 |
| chr1 | 145696694 | G | A | CD160,NBPF20,NBPF10 | intronic | . | rs2231375 |
| chr1 | 26646574 | G | A | CD52 | intronic | . | rs112252579 |
| chr1 | 26646726 | A | G | CD52 | exonic | . | rs1071849 |
| chr1 | 26646730 | A | G | CD52 | exonic | . | rs17645 |
| chr1 | 196642233 | G | A | CFH | exonic | . | rs800292 |
| chr1 | 196682947 | G | A | CFH | exonic | . | rs2274700 |
| chr1 | 196654324 | A | C | CFH | exonic | . | rs1061147 |
| chr1 | 196659237 | C | T | CFH | exonic | . | rs1061170 |
| chr1 | 196642072 | C | T | CFH | intronic | . | rs551397 |
| chr1 | 196658497 | G | T | CFH | intronic | . | rs482934 |
| chr1 | 196695742 | A | G | CFH | exonic | . | rs3753396 |
| chr1 | 196709774 | G | T | CFH | exonic | . | rs1065489 |
| chr1 | 196801078 | A | T | CFHR1 | exonic | . | rs414628 |
| chr1 | 196801042 | G | T | CFHR1 | exonic | . | rs4230 |
| chr1 | 196797123 | C | T | CFHR1 | intronic | . | rs677961 |
| chr1 | 196801190 | C | A | CFHR1 | UTR3 | NM_002113:c.*61C>A | rs390679 |
| chr1 | 196799691 | G | A | CFHR1 | exonic | . | rs12406079 |
| chr1 | 196797238 | C | T | CFHR1 | exonic | . | rs425757 |
| chr1 | 196797244 | C | G | CFHR1 | exonic | . | rs113811987 |
| chr1 | 196799746 | C | G | CFHR1 | exonic | . | rs147253539 |
| chr1 | 196797493 | T | C | CFHR1 | intronic | . | rs28369298 |
| chr1 | 196926956 | C | T | CFHR2 | intronic | . | rs3748556 |
| chr1 | 196926973 | C | A | CFHR2 | intronic | . | rs3748555 |
| chr1 | 196758778 | G | A | CFHR3 | intronic | . | rs384032 |
| chr1 | 196749097 | C | T | CFHR3 | exonic | . | rs61737525 |
| chr1 | 196749149 | G | A | CFHR3 | intronic | . | rs73073579 |
| chr1 | 209905734 | G | C | HSD11B1 | intronic | . | rs932335 |
| chr1 | 209880259 | T | G | HSD11B1 | intronic | . | rs12086634 |
| chr1 | 120057158 | C | T | HSD3B1 | exonic | . | rs6203 |
| chr1 | 119964255 | T | C | HSD3B2 | intronic | . | rs58154933 |
| chr1 | 119957883 | T | G | HSD3B2 | intronic | . | rs714748 |
| chr1 | 120110768 | C | T | HSD3BP4 | ncRNA_exonic | . | rs12746459 |
| chr1 | 120110779 | C | T | HSD3BP4 | ncRNA_exonic | . | rs71519737 |
| chr1 | 120110747 | A | G | HSD3BP4 | ncRNA_exonic | . | rs12727820 |
| chr1 | 120110755 | T | C | HSD3BP4 | ncRNA_exonic | . | rs12731874 |
| chr1 | 120110708 | G | T | HSD3BP4 | ncRNA_exonic | . | rs12745178 |
| chr1 | 120110730 | G | C | HSD3BP4 | ncRNA_exonic | . | rs781075060 |
| chr1 | 120110783 | A | G | HSD3BP4 | ncRNA_exonic | . | rs71519739 |
| chr1 | 120110685 | G | A | HSD3BP4 | ncRNA_exonic | . | rs12745167 |
| chr1 | 206944952 | T | G | IL10 | intronic | . | rs3021094 |
| chr1 | 206944645 | T | C | IL10 | intronic | . | rs1518111 |
| chr1 | 206944861 | A | C | IL10 | intronic | . | rs1518110 |
| chr1 | 206944233 | A | G | IL10 | intronic | . | rs1554286 |
| chr1 | 206941945 | A | G | IL10 | UTR3 | NM_000572:c.*36T>C | rs5743628 |
| chr1 | 166899807 | T | C | ILDR2 | intronic | . | rs2013526 |
| chr1 | 145277317 | G | A | NBPF20,NBPF9,NOTCH2NL | intronic | . | rs150961562 |
| chr1 | 145277311 | T | C | NBPF20,NBPF9,NOTCH2NL | intronic | . | rs148585489 |
| chr1 | 145277327 | C | A | NBPF20,NBPF9,NOTCH2NL | intronic | . | rs145481915 |
| chr1 | 25234062 | T | G | RUNX3 | intronic | . | rs34878696 |
| chr1 | 25256444 | G | A | RUNX3 | UTR5 | NM_004350:c.-85C>T | rs71514255 |
| chr1 | 25291010 | A | T | RUNX3 | exonic | . | rs6672420 |
| chr1 | 25254317 | C | T | RUNX3 | intronic | . | rs7517302 |
| chr1 | 92224067 | C | T | TGFBR3 | intronic | . | rs3738441 |
| chr1 | 92149277 | C | T | TGFBR3 | UTR3 | NM_001195683:c.*19G>A;NM_001195684:c.*19G>A;NM_003243:c.*19G>A | rs1131243 |
| chr1 | 92163786 | G | T | TGFBR3 | intronic | . | rs2296621 |
| chr1 | 92177938 | A | G | TGFBR3 | exonic | . | rs1805113 |
| chr1 | 92184814 | G | T | TGFBR3 | intronic | . | rs7524066 |
| chr1 | 92262874 | T | C | TGFBR3 | exonic | . | rs2810904 |
| chr1 | 92178260 | A | G | TGFBR3 | intronic | . | rs1613413 |
| chr1 | 92149139 | C | T | TGFBR3 | UTR3 | NM_001195683:c.*157G>A;NM_001195684:c.*157G>A;NM_003243:c.*157G>A | rs1805115 |
| chr1 | 92224347 | G | A | TGFBR3 | intronic | . | rs11165441 |
| chr1 | 92195601 | G | A | TGFBR3 | intronic | . | rs10783002 |
| chr1 | 92174415 | G | A | TGFBR3 | intronic | . | rs2038931 |
| chr1 | 92174260 | A | G | TGFBR3 | exonic | . | rs284878 |
| chr1 | 92327126 | C | T | TGFBR3 | UTR5 | NM_001195683:c.-38G>A;NM_001195684:c.-38G>A;NM_003243:c.-38G>A | rs1805109 |
| chr1 | 92327045 | G | A | TGFBR3 | exonic | . | rs1805110 |
| chr1 | 92200593 | A | G | TGFBR3 | intronic | . | rs11165376 |
| chr1 | 92185657 | C | T | TGFBR3 | exonic | . | rs1805112 |
| chr1 | 92161515 | T | A | TGFBR3 | intronic | . | rs2253316 |
| chr1 | 92178259 | C | T | TGFBR3 | intronic | . | rs1750641 |
| chr1 | 92200597 | G | A | TGFBR3 | intronic | . | rs12124904 |
| chr1 | 92200634 | C | T | TGFBR3 | intronic | . | rs11165377 |
| chr1 | 92200382 | T | C | TGFBR3 | exonic | . | rs2306888 |
| chr1 | 92181670 | C | A | TGFBR3 | intronic | . | rs2029354 |
| chr1 | 92181678 | T | G | TGFBR3 | intronic | . | rs2029355 |
| chr1 | 92184744 | C | T | TGFBR3 | intronic | . | rs4658261 |
| chr1 | 2488153 | A | G | TNFRSF14 | exonic | . | rs4870 |
| chr1 | 2496649 | C | G | TNFRSF14 | UTR3 | NM_001297605:c.*2091C>G;NM_003820:c.*1937C>G | rs7544646 |
| chr1 | 2491306 | G | A | TNFRSF14 | exonic | . | rs2234163 |
| chr1 | 2491205 | C | T | TNFRSF14 | intronic | . | rs2234161 |
| chr1 | 2496741 | C | T | TNFRSF14 | UTR3 | NM_001297605:c.*2183C>T;NM_003820:c.*2029C>T | rs144730541 |
| chr1 | 2494785 | G | A | TNFRSF14 | UTR3 | NM_001297605:c.*227G>A;NM_003820:c.*73G>A | rs8725 |
| chr1 | 2496653 | A | G | TNFRSF14 | UTR3 | NM_001297605:c.*2095A>G;NM_003820:c.*1941A>G | rs7515633 |
| chr1 | 175615257 | C | T | TNR | intronic | . | rs10913045 |
| chr1 | 101197183 | G | A | VCAM1 | intronic | . | rs3176870 |
| chr1 | 101190173 | C | T | VCAM1 | splicing | NM_001078:exon4:c.662-7C>T;NM_001199834:exon4:c.476-7C>T;NM_080682:exon4:c.662-7C>T | rs2392221 |
| chr1 | 101199979 | A | G | VCAM1 | intronic | . | rs3176875 |
| chr1 | 101203827 | G | A | VCAM1 | exonic | . | rs3176879 |
| chr10 | 101602004 | T | C | ABCC2 | intronic | . | rs3758395 |
| chr10 | 101589925 | G | A | ABCC2 | intronic | . | rs4148395 |
| chr10 | 101567426 | A | G | ABCC2 | intronic | . | rs2073337 |
| chr10 | 101610306 | C | T | ABCC2 | intronic | . | rs3824610 |
| chr10 | 101563815 | G | A | ABCC2 | exonic | . | rs2273697 |
| chr10 | 101553259 | C | T | ABCC2 | intronic | . | rs2804400 |
| chr10 | 101542578 | C | T | ABCC2 | UTR5 | NM_000392:c.-24C>T | rs717620 |
| chr10 | 101576960 | T | C | ABCC2 | intronic | . | rs3740073 |
| chr10 | 101610723 | A | G | ABCC2 | intronic | . | rs3740063 |
| chr10 | 101604207 | C | T | ABCC2 | exonic | . | rs3740066 |
| chr10 | 101591944 | T | C | ABCC2 | intronic | . | rs4148396 |
| chr10 | 101603781 | C | G | ABCC2 | intronic | . | rs3740067 |
| chr10 | 101558746 | G | T | ABCC2 | intronic | . | rs2756109 |
| chr10 | 96448129 | A | G | CYP2C18 | intronic | . | rs1926706 |
| chr10 | 96493058 | C | T | CYP2C18 | exonic | . | rs1126545 |
| chr10 | 96494912 | C | T | CYP2C18 | intronic | . | rs79429965 |
| chr10 | 96495232 | C | T | CYP2C18 | UTR3 | NM_000772:c.*31C>T;NM_001128925:c.*31C>T | rs2860840 |
| chr10 | 96484233 | C | T | CYP2C18 | exonic | . | rs76498052 |
| chr10 | 96447562 | T | A | CYP2C18 | exonic | . | rs41291550 |
| chr10 | 96495284 | G | A | CYP2C18 | UTR3 | NM_000772:c.*83G>A;NM_001128925:c.*83G>A | rs1042192 |
| chr10 | 96580202 | C | G | CYP2C19 | intronic | . | rs4417205 |
| chr10 | 96541616 | G | A | CYP2C19 | exonic | . | rs4244285 |
| chr10 | 96535124 | A | G | CYP2C19 | intronic | . | rs12769205 |
| chr10 | 96602622 | C | T | CYP2C19 | exonic | . | rs3758580 |
| chr10 | 96602623 | G | A | CYP2C19 | exonic | . | rs3758581 |
| chr10 | 96609568 | T | C | CYP2C19 | intronic | . | rs4917623 |
| chr10 | 96522561 | T | C | CYP2C19 | exonic | . | rs17885098 |
| chr10 | 96534768 | G | A | CYP2C19 | intronic | . | rs17878649 |
| chr10 | 96609775 | A | C | CYP2C19 | exonic | . | rs17886522 |
| chr10 | 96540410 | G | A | CYP2C19 | exonic | . | rs4986893 |
| chr10 | 96805371 | C | T | CYP2C8 | intronic | . | rs1891071 |
| chr10 | 96802598 | A | T | CYP2C8 | intronic | . | rs2275620 |
| chr10 | 96827178 | T | C | CYP2C8 | intronic | . | rs2275622 |
| chr10 | 96798548 | C | T | CYP2C8 | intronic | . | rs1934951 |
| chr10 | 96824883 | T | C | CYP2C8 | intronic | . | rs3752988 |
| chr10 | 96796861 | G | A | CYP2C8 | UTR3 | NM_000770:c.*24C>T;NM_001198853:c.*24C>T;NM_001198854:c.*24C>T;NM_001198855:c.*24C>T | rs1058932 |
| chr10 | 96818362 | A | G | CYP2C8 | intronic | . | rs11572101 |
| chr10 | 96824738 | A | T | CYP2C8 | intronic | . | rs7098376 |
| chr10 | 96824406 | C | T | CYP2C8 | intronic | . | rs11572093 |
| chr10 | 96748495 | A | T | CYP2C9 | intronic | . | rs1934969 |
| chr10 | 96745984 | A | T | CYP2C9 | intronic | . | rs9332230 |
| chr10 | 96741053 | A | C | CYP2C9 | exonic | . | rs1057910 |
| chr10 | 96748737 | A | T | CYP2C9 | exonic | . | rs1057911 |
| chr10 | 96707471 | G | C | CYP2C9 | intronic | . | rs9332127 |
| chr10 | 6067688 | A | G | IL2RA | intronic | . | rs2025345 |
| chr10 | 6062013 | G | A | IL2RA | intronic | . | rs2274036 |
| chr10 | 6059898 | C | A | IL2RA | intronic | . | rs7899538 |
| chr10 | 6061781 | T | C | IL2RA | intronic | . | rs10752175 |
| chr10 | 6066200 | C | G | IL2RA | splicing | NM_000417:exon4:c.367+7G>C | rs11256369 |
| chr10 | 6066195 | T | A | IL2RA | intronic | . | rs12358961 |
| chr10 | 6063319 | C | T | IL2RA | intronic | . | rs7093069 |
| chr10 | 49633875 | T | A | MAPK8 | intronic | . | rs201968887 |
| chr10 | 49617960 | C | T | MAPK8 | exonic | . | rs55740545 |
| chr10 | 49618231 | G | A | MAPK8 | intronic | . | rs73309917 |
| chr10 | 49609892 | T | G | MAPK8 | intronic | . | rs74808108 |
| chr10 | 49635059 | A | G | MAPK8 | intronic | . | rs76341585 |
| chr10 | 49635086 | C | T | MAPK8 | intronic | . | rs80180843 |
| chr10 | 114710876 | C | T | TCF7L2 | intronic | . | rs78277257 |
| chr10 | 114849353 | C | T | TCF7L2 | intronic | . | rs10749127 |
| chr10 | 114925369 | C | A | TCF7L2 | exonic | . | rs77961654 |
| chr10 | 114724473 | A | G | TCF7L2 | intronic | . | rs11196171 |
| chr10 | 114849399 | C | T | TCF7L2 | intronic | . | rs4917644 |
| chr10 | 60095105 | G | T | UBE2D1 | intronic | . | rs112660736 |
| chr10 | 60127639 | G | T | UBE2D1 | intronic | . | rs74377516 |
| chr10 | 60123486 | A | G | UBE2D1 | intronic | . | rs73288305 |
| chr10 | 60127838 | G | A | UBE2D1 | intronic | . | rs75362994 |
| chr11 | 116661826 | T | C | APOA5 | intronic | . | rs2072560 |
| chr11 | 116662579 | C | T | APOA5 | UTR5 | NM_001166598:c.-3G>A;NM_052968:c.-3G>A | rs651821 |
| chr11 | 116661488 | C | T | APOA5 | exonic | . | rs3135507 |
| chr11 | 116660768 | G | A | APOA5 | UTR3 | NM_001166598:c.*76C>T;NM_052968:c.*76C>T | rs34089864 |
| chr11 | 116703671 | G | T | APOC3 | UTR3 | NM_000040:c.*71G>T | rs4225 |
| chr11 | 116701153 | T | G | APOC3 | intronic | . | rs2070668 |
| chr11 | 116701674 | T | A | APOC3 | intronic | . | rs2070666 |
| chr11 | 116701535 | T | C | APOC3 | exonic | . | rs4520 |
| chr11 | 116701669 | G | A | APOC3 | intronic | . | rs2070667 |
| chr11 | 116701122 | C | G | APOC3 | intronic | . | rs2070669 |
| chr11 | 116703640 | G | C | APOC3 | UTR3 | NM_000040:c.*40G>C | rs5128 |
| chr11 | 35243434 | C | G | CD44 | intronic | . | rs55927167 |
| chr11 | 35208251 | A | G | CD44 | intronic | . | rs3736812 |
| chr11 | 35198108 | A | G | CD44 | intronic | . | rs4756196 |
| chr11 | 35244058 | A | G | CD44 | UTR3 | NM_001202557:c.*133A>G | rs7116432 |
| chr11 | 35240789 | T | A | CD44 | intronic | . | rs12577824 |
| chr11 | 35226155 | A | G | CD44 | exonic | . | rs9666607 |
| chr11 | 35208638 | G | A | CD44 | intronic | . | rs61881658 |
| chr11 | 35201982 | C | T | CD44 | intronic | . | rs76393888 |
| chr11 | 35223158 | T | C | CD44 | intronic | . | rs11033025 |
| chr11 | 35201842 | C | T | CD44 | exonic | . | rs1071695 |
| chr11 | 614864 | C | T | IRF7 | exonic | . | rs1061501 |
| chr11 | 102666316 | T | C | MMP1 | exonic | . | rs470558 |
| chr11 | 102649856 | T | C | MMP10 | intronic | . | rs470263 |
| chr11 | 102643756 | C | G | MMP10 | intronic | . | rs142994202 |
| chr11 | 60230531 | C | T | MS4A1 | exonic | . | rs2070770 |
| chr11 | 60230722 | G | T | MS4A1 | intronic | . | rs10792278 |
| chr11 | 60235683 | G | T | MS4A1 | intronic | . | rs1941031 |
| chr11 | 132090927 | G | A | NTM | intronic | . | rs2335467 |
| chr11 | 132091073 | C | T | NTM | intronic | . | rs921269 |
| chr11 | 74883577 | G | A | SLCO2B1 | exonic | . | rs12422149 |
| chr11 | 74880370 | G | A | SLCO2B1 | exonic | . | rs35199625 |
| chr11 | 74880856 | C | A | SLCO2B1 | intronic | . | rs7125268 |
| chr11 | 74862391 | A | G | SLCO2B1 | UTR5 | NM_001145212:c.-36A>G;NM_007256:c.-36A>G | rs1944612 |
| chr11 | 74907582 | C | T | SLCO2B1 | exonic | . | rs2306168 |
| chr11 | 74907900 | A | C | SLCO2B1 | intronic | . | rs3781725 |
| chr11 | 74862356 | T | C | SLCO2B1 | UTR5 | NM_001145212:c.-71T>C;NM_007256:c.-71T>C | rs2851069 |
| chr11 | 74907865 | A | G | SLCO2B1 | intronic | . | rs3781724 |
| chr11 | 74877004 | G | A | SLCO2B1 | splicing | NM_001145211:exon4:c.382+10G>A;NM_007256:exon4:c.448+10G>A | rs3740839 |
| chr11 | 74877153 | A | T | SLCO2B1 | intronic | . | rs867627 |
| chr11 | 102668702 | T | C | WTAPP1 | ncRNA_intronic | . | rs470358 |
| chr11 | 102663482 | A | T | WTAPP1 | ncRNA_intronic | . | rs1144391 |
| chr11 | 102661099 | T | C | WTAPP1 | ncRNA_intronic | . | rs470215 |
| chr11 | 102661595 | A | G | WTAPP1 | ncRNA_intronic | . | rs470747 |
| chr11 | 102661080 | G | A | WTAPP1 | ncRNA_intronic | . | rs2239008 |
| chr11 | 102661665 | G | A | WTAPP1 | ncRNA_intronic | . | rs1938901 |
| chr11 | 102661276 | T | G | WTAPP1 | ncRNA_intronic | . | rs2071231 |
| chr11 | 102666043 | A | G | WTAPP1 | ncRNA_intronic | . | rs491152 |
| chr11 | 102663503 | T | C | WTAPP1 | ncRNA_intronic | . | rs7125062 |
| chr12 | 112230036 | G | A | ALDH2 | intronic | . | rs4646777 |
| chr12 | 112235783 | C | A | ALDH2 | intronic | . | rs4646778 |
| chr12 | 112221055 | C | T | ALDH2 | exonic | . | rs13306164 |
| chr12 | 112241552 | T | A | ALDH2 | intronic | . | rs2106696 |
| chr12 | 112230019 | G | C | ALDH2 | intronic | . | rs4646776 |
| chr12 | 112241766 | G | A | ALDH2 | exonic | . | rs671 |
| chr12 | 32459070 | T | G | BICD1 | intronic | . | rs41521147 |
| chr12 | 32490764 | G | A | BICD1 | intronic | . | rs2270786 |
| chr12 | 32446901 | T | C | BICD1 | intronic | . | rs261878 |
| chr12 | 32530385 | C | G | BICD1 | intronic | . | rs7298319 |
| chr12 | 32491626 | G | A | BICD1 | intronic | . | rs1471909 |
| chr12 | 32490850 | C | G | BICD1 | intronic | . | rs1057779 |
| chr12 | 32490369 | G | A | BICD1 | intronic | . | rs2388987 |
| chr12 | 32481021 | C | A | BICD1 | exonic | . | rs75160526 |
| chr12 | 32490275 | G | A | BICD1 | intronic | . | rs2388989 |
| chr12 | 32487644 | T | A | BICD1 | intronic | . | rs10844188 |
| chr12 | 32481093 | G | C | BICD1 | exonic | . | rs3748275 |
| chr12 | 32530580 | G | A | BICD1 | UTR3 | NM_001003398:c.*135G>A;NM_001714:c.*19G>A | rs11051966 |
| chr12 | 32490348 | G | A | BICD1 | intronic | . | rs2388988 |
| chr12 | 32490849 | G | A | BICD1 | intronic | . | rs7309590 |
| chr12 | 32481577 | A | G | BICD1 | intronic | . | rs3748276 |
| chr12 | 32490375 | C | G | BICD1 | intronic | . | rs4604975 |
| chr12 | 32369071 | A | G | BICD1 | intronic | . | rs161975 |
| chr12 | 21330022 | G | A | SLCO1B1 | intronic | . | rs4149046 |
| chr12 | 21331987 | C | T | SLCO1B1 | intronic | . | rs2291076 |
| chr12 | 21331599 | T | C | SLCO1B1 | exonic | . | rs4149057 |
| chr12 | 21331549 | T | C | SLCO1B1 | exonic | . | rs4149056 |
| chr12 | 21377559 | G | C | SLCO1B1 | intronic | . | rs4149080 |
| chr12 | 21331625 | C | T | SLCO1B1 | exonic | . | rs2291075 |
| chr12 | 21378021 | G | A | SLCO1B1 | intronic | . | rs4149081 |
| chr12 | 21327740 | C | A | SLCO1B1 | intronic | . | rs4149036 |
| chr12 | 21369883 | C | G | SLCO1B1 | intronic | . | rs4149070 |
| chr12 | 21353872 | A | G | SLCO1B1 | intronic | . | rs4149066 |
| chr12 | 21325949 | A | G | SLCO1B1 | intronic | . | rs2291074 |
| chr12 | 21353911 | C | G | SLCO1B1 | intronic | . | rs4149067 |
| chr12 | 21329996 | A | T | SLCO1B1 | intronic | . | rs4149044 |
| chr12 | 21392290 | T | C | SLCO1B1 | UTR3 | NM_006446:c.*167T>C | rs4149085 |
| chr12 | 21369964 | T | C | SLCO1B1 | intronic | . | rs4149071 |
| chr12 | 21369985 | G | A | SLCO1B1 | intronic | . | rs4149072 |
| chr12 | 21325814 | T | G | SLCO1B1 | intronic | . | rs2291073 |
| chr12 | 21054369 | G | A | SLCO1B3 | exonic | . | rs3764006 |
| chr12 | 21054918 | T | C | SLCO1B3 | intronic | . | rs2082518 |
| chr12 | 21030672 | A | T | SLCO1B3 | intronic | . | rs4149139 |
| chr12 | 21011313 | A | C | SLCO1B3 | intronic | . | rs149520180 |
| chr12 | 21051489 | C | T | SLCO1B3 | intronic | . | rs4149153 |
| chr12 | 21015815 | T | G | SLCO1B3 | intronic | . | rs16923270 |
| chr12 | 21015864 | C | G | SLCO1B3 | intronic | . | rs71583718 |
| chr12 | 21028093 | T | C | SLCO1B3 | intronic | . | rs3829311 |
| chr12 | 21011310 | T | C | SLCO1B3 | intronic | . | rs4149116 |
| chr12 | 21034017 | A | T | SLCO1B3 | intronic | . | rs4149144 |
| chr12 | 21011581 | G | A | SLCO1B3 | intronic | . | rs4149118 |
| chr12 | 21036326 | G | T | SLCO1B3 | intronic | . | rs4149151 |
| chr12 | 21068915 | C | T | SLCO1B3 | intronic | . | rs78862986 |
| chr12 | 21015610 | A | G | SLCO1B3 | intronic | . | rs1036261 |
| chr12 | 21013948 | C | T | SLCO1B3 | splicing | NM_019844:exon6:c.360-3C>T | rs3764009 |
| chr12 | 21036411 | A | G | SLCO1B3 | exonic | . | rs2053098 |
| chr12 | 21011480 | T | G | SLCO1B3 | exonic | . | rs4149117 |
| chr12 | 21032242 | T | G | SLCO1B3 | intronic | . | rs4149142 |
| chr12 | 21015760 | G | A | SLCO1B3 | exonic | . | rs7311358 |
| chr12 | 21196533 | C | T | SLCO1B7 | intronic | . | rs76323373 |
| chr12 | 21243001 | C | T | SLCO1B7 | exonic | . | rs73241801 |
| chr12 | 21220349 | A | T | SLCO1B7 | intronic | . | rs1910163 |
| chr12 | 21229693 | C | T | SLCO1B7 | intronic | . | rs7315334 |
| chr12 | 21207346 | T | C | SLCO1B7 | intronic | . | rs7312369 |
| chr12 | 21176247 | A | G | SLCO1B7 | intronic | . | rs1546307 |
| chr13 | 40101829 | C | T | LHFP | intronic | . | rs9576835 |
| chr13 | 40101913 | G | C | LHFP | intronic | . | rs4325425 |
| chr13 | 40101740 | C | T | LHFP | intronic | . | rs730249 |
| chr14 | 24839083 | G | C | NFATC4 | exonic | . | rs2229309 |
| chr14 | 24845154 | C | A | NFATC4 | intronic | . | rs12880769 |
| chr14 | 24843699 | C | T | NFATC4 | intronic | . | rs12890614 |
| chr14 | 24843620 | T | C | NFATC4 | exonic | . | rs2295298 |
| chr14 | 24845841 | T | C | NFATC4 | exonic | . | rs7149586 |
| chr14 | 24838621 | C | T | NFATC4 | intronic | . | rs1955915 |
| chr14 | 24841517 | A | G | NFATC4 | intronic | . | rs10141527 |
| chr14 | 24846757 | A | G | NFATC4 | UTR3 | NM_001136022:c.*608A>G;NM_001288802:c.*608A>G | rs2243891 |
| chr14 | 24839165 | C | T | NFATC4 | exonic | . | rs2228233 |
| chr14 | 24837668 | G | C | NFATC4 | intronic | . | rs45565135 |
| chr14 | 24845402 | G | T | NFATC4 | intronic | . | rs10141896 |
| chr14 | 24836815 | C | G | NFATC4 | intronic | . | rs28365903 |
| chr14 | 24846961 | G | T | NFATC4 | UTR3 | NM_001136022:c.*812G>T;NM_001198965:c.*50G>T;NM_001198966:c.*50G>T;NM_001198967:c.*50G>T;NM_001288802:c.*812G>T;NM_004554:c.*50G>T | rs10362 |
| chr14 | 76429555 | C | T | TGFB3 | intronic | . | rs3917201 |
| chr14 | 76432136 | T | C | TGFB3 | intronic | . | rs3917187 |
| chr15 | 23000272 | A | G | CYFIP1 | intronic | . | rs999842 |
| chr15 | 68349893 | A | G | PIAS1 | intronic | . | rs1489598 |
| chr15 | 61076591 | A | G | RORA | intronic | . | rs877228 |
| chr15 | 67457850 | G | A | SMAD3 | intronic | . | rs2289259 |
| chr15 | 67358478 | G | A | SMAD3 | UTR5 | NM_005902:c.-15G>A | rs1061427 |
| chr15 | 67459013 | C | T | SMAD3 | intronic | . | rs7179893 |
| chr15 | 67457335 | A | G | SMAD3 | exonic | . | rs1065080 |
| chr15 | 67457485 | G | C | SMAD3 | intronic | . | rs2289261 |
| chr15 | 67476952 | G | T | SMAD3 | intronic | . | rs2289791 |
| chr15 | 67476970 | T | C | SMAD3 | intronic | . | rs2289790 |
| chr16 | 10205430 | A | G | GRIN2A | intronic | . | rs7190342 |
| chr16 | 10205412 | G | T | GRIN2A | intronic | . | rs7188171 |
| chr16 | 10206652 | G | A | GRIN2A | intronic | . | rs1900718 |
| chr16 | 10205788 | A | G | GRIN2A | intronic | . | rs11646734 |
| chr16 | 10205656 | T | A | GRIN2A | intronic | . | rs7195607 |
| chr16 | 10205824 | C | G | GRIN2A | intronic | . | rs11641504 |
| chr16 | 10205691 | G | C | GRIN2A | intronic | . | rs34393853 |
| chr16 | 10206426 | G | A | GRIN2A | intronic | . | rs34819762 |
| chr16 | 10205326 | C | T | GRIN2A | intronic | . | rs7189495 |
| chr16 | 85948098 | G | A | IRF8 | exonic | . | rs17444416 |
| chr16 | 85945076 | T | C | IRF8 | intronic | . | rs2292980 |
| chr16 | 85942496 | T | C | IRF8 | intronic | . | rs305079 |
| chr16 | 85953909 | A | G | IRF8 | intronic | . | rs35593615 |
| chr16 | 85953682 | G | C | IRF8 | intronic | . | rs16940005 |
| chr16 | 30134251 | C | G | MAPK3 | intronic | . | rs80260207 |
| chr16 | 55527298 | G | A | MMP2 | intronic | . | rs243843 |
| chr16 | 55525913 | G | C | MMP2 | intronic | . | rs243846 |
| chr16 | 55519607 | C | T | MMP2 | exonic | . | rs1053605 |
| chr16 | 55527026 | A | T | MMP2 | intronic | . | rs11640428 |
| chr16 | 55532458 | T | C | MMP2 | intronic | . | rs2287076 |
| chr16 | 55522752 | A | G | MMP2 | intronic | . | rs138012091 |
| chr16 | 55527113 | G | A | MMP2 | exonic | . | rs2287074 |
| chr16 | 55523782 | G | C | MMP2 | intronic | . | rs12599775 |
| chr16 | 55532120 | G | A | MMP2 | intronic | . | rs2287075 |
| chr16 | 55523705 | T | C | MMP2 | exonic | . | rs243849 |
| chr16 | 55513331 | G | C | MMP2 | UTR5 | NM_004530:c.-61G>C | rs2287073 |
| chr16 | 55530772 | T | G | MMP2 | intronic | . | rs243838 |
| chr16 | 55526876 | G | A | MMP2 | intronic | . | rs243844 |
| chr16 | 55536687 | A | G | MMP2 | splicing | NM_001127891:exon12:c.1620-4A>G;NM_001302508:exon12:c.1542-4A>G;NM_001302509:exon12:c.1542-4A>G;NM_001302510:exon12:c.1542-4A>G;NM_004530:exon12:c.1770-4A>G | rs243834 |
| chr16 | 55536622 | C | T | MMP2 | intronic | . | rs243835 |
| chr16 | 55539191 | C | G | MMP2 | intronic | . | rs243832 |
| chr16 | 55516767 | G | A | MMP2 | intronic | . | rs1030868 |
| chr16 | 55519535 | G | C | MMP2 | exonic | . | rs1132896 |
| chr16 | 55530762 | G | T | MMP2 | intronic | . | rs171498 |
| chr16 | 55517162 | A | G | MMP2 | intronic | . | rs1477017 |
| chr16 | 55513623 | C | T | MMP2 | intronic | . | rs243862 |
| chr16 | 55536727 | C | T | MMP2 | exonic | . | rs14070 |
| chr16 | 55523902 | G | T | MMP2 | intronic | . | rs17859922 |
| chr16 | 55519832 | T | G | MMP2 | intronic | . | rs111278338 |
| chr16 | 55519701 | C | T | MMP2 | intronic | . | rs17859889 |
| chr16 | 55531030 | C | T | MMP2 | intronic | . | rs17859975 |
| chr16 | 1370716 | C | T | UBE2I | intronic | . | rs142273742 |
| chr16 | 1374656 | A | G | UBE2I | intronic | . | rs761060 |
| chr16 | 1364365 | A | G | UBE2I | exonic | . | rs4610 |
| chr16 | 1374818 | A | G | UBE2I | UTR3 | NM_003345:c.*24A>G;NM_194259:c.*24A>G;NM_194260:c.*24A>G;NM_194261:c.*24A>G | rs8063 |
| chr16 | 1374524 | A | G | UBE2I | intronic | . | rs761059 |
| chr16 | 1370682 | G | A | UBE2I | intronic | . | rs79005361 |
| chr17 | 61574492 | G | A | ACE | splicing | NM_000789:exon25:c.3692-6G>A;NM_001178057:exon13:c.1847-6G>A;NM_152830:exon14:c.1970-6G>A | rs4363 |
| chr17 | 61573761 | T | C | ACE | exonic | . | rs4362 |
| chr17 | 61556298 | C | G | ACE | intronic | . | rs4295 |
| chr17 | 61559923 | C | T | ACE | exonic | . | rs4309 |
| chr17 | 61563661 | C | A | ACE | intronic | . | rs4330 |
| chr17 | 61562774 | T | C | ACE | intronic | . | rs4321 |
| chr17 | 61562309 | C | T | ACE | exonic | . | rs4316 |
| chr17 | 61562553 | G | A | ACE | intronic | . | rs4320 |
| chr17 | 61564052 | A | G | ACE | exonic | . | rs4331 |
| chr17 | 61564281 | T | C | ACE | intronic | . | rs4332 |
| chr17 | 61564522 | T | C | ACE | intronic | . | rs4333 |
| chr17 | 61565990 | G | C | ACE | intronic | . | rs4341 |
| chr17 | 61565998 | A | C | ACE | intronic | . | rs4342 |
| chr17 | 61566031 | G | A | ACE | exonic | . | rs4343 |
| chr17 | 61560763 | T | C | ACE | intronic | . | rs4311 |
| chr17 | 28935645 | G | A | LRRC37BP1 | ncRNA_intronic | . | rs216416 |
| chr17 | 28934549 | C | A | LRRC37BP1 | ncRNA_intronic | . | rs75136574 |
| chr17 | 28934550 | C | A | LRRC37BP1 | ncRNA_intronic | . | rs77544470 |
| chr17 | 28934548 | T | G | LRRC37BP1 | ncRNA_intronic | . | rs79607958 |
| chr17 | 72935809 | T | A | OTOP3 | intronic | . | rs1388511 |
| chr17 | 72935805 | G | A | OTOP3 | intronic | . | rs1388512 |
| chr17 | 78877735 | G | A | RPTOR | intronic | . | rs868432 |
| chr18 | 44663687 | C | T | HDHD2 | intronic | . | rs79420880 |
| chr18 | 77246978 | G | A | NFATC1 | intronic | . | rs754096 |
| chr18 | 77246406 | T | G | NFATC1 | exonic | . | rs754093 |
| chr18 | 77227476 | A | G | NFATC1 | exonic | . | rs25656 |
| chr18 | 77221264 | C | T | NFATC1 | intronic | . | rs2304739 |
| chr18 | 77221278 | G | A | NFATC1 | intronic | . | rs56112793 |
| chr18 | 77221207 | C | A | NFATC1 | intronic | . | rs2304738 |
| chr18 | 77211175 | T | C | NFATC1 | intronic | . | rs2290154 |
| chr18 | 77211764 | T | C | NFATC1 | exonic | . | rs15350 |
| chr18 | 77209015 | A | G | NFATC1 | intronic | . | rs2278801 |
| chr18 | 77287385 | T | C | NFATC1 | intronic | . | rs451691 |
| chr18 | 77208922 | C | T | NFATC1 | exonic | . | rs25657 |
| chr18 | 77171061 | T | G | NFATC1 | exonic | . | rs2230112 |
| chr18 | 77156174 | G | A | NFATC1 | UTR5 | NM_001278669:c.-51G>A;NM_001278670:c.-51G>A;NM_006162:c.-51G>A;NM_172388:c.-52638G>A;NM_172390:c.-51G>A | rs139536097 |
| chr18 | 77156197 | C | T | NFATC1 | UTR5 | NM_001278669:c.-28C>T;NM_001278670:c.-28C>T;NM_006162:c.-28C>T;NM_172388:c.-52615C>T;NM_172390:c.-28C>T | rs62096862 |
| chr18 | 44426877 | C | T | PIAS2 | intronic | . | rs644731 |
| chr18 | 44395368 | T | C | PIAS2 | intronic | . | rs3737448 |
| chr18 | 44470706 | G | A | PIAS2 | exonic | . | rs113887072 |
| chr18 | 48575389 | T | C | SMAD4 | intronic | . | rs2276163 |
| chr18 | 48577782 | G | C | SMAD4 | intronic | . | rs7229678 |
| chr19 | 45412079 | C | T | APOE | exonic | . | rs7412 |
| chr19 | 45409167 | C | G | APOE | exonic | . | rs440446 |
| chr19 | 45411941 | T | C | APOE | exonic | . | rs429358 |
| chr19 | 45410002 | G | A | APOE | intronic | . | rs769449 |
| chr19 | 6718078 | T | G | C3 | intronic | . | rs2547438 |
| chr19 | 6693163 | G | A | C3 | intronic | . | rs10414623 |
| chr19 | 6696699 | G | A | C3 | intronic | . | rs2253756 |
| chr19 | 6696597 | G | A | C3 | splicing | NM_000064:exon23:c.2863+7C>T | rs2287845 |
| chr19 | 6696557 | C | G | C3 | intronic | . | rs2287846 |
| chr19 | 6696691 | G | T | C3 | intronic | . | rs2355315 |
| chr19 | 6697829 | G | T | C3 | intronic | . | rs366510 |
| chr19 | 6702157 | C | G | C3 | exonic | . | rs428453 |
| chr19 | 6702246 | T | C | C3 | intronic | . | rs432823 |
| chr19 | 6718534 | T | C | C3 | intronic | . | rs2250656 |
| chr19 | 6696789 | G | A | C3 | intronic | . | rs2355316 |
| chr19 | 6696835 | G | A | C3 | intronic | . | rs408004 |
| chr19 | 6679511 | C | T | C3 | splicing | NM_000064:exon38:c.4457-4G>A | rs2277984 |
| chr19 | 6693571 | C | T | C3 | intronic | . | rs11085194 |
| chr19 | 6696342 | A | G | C3 | intronic | . | rs2287848 |
| chr19 | 6677989 | G | A | C3 | exonic | . | rs17030 |
| chr19 | 6679563 | T | C | C3 | intronic | . | rs2277983 |
| chr19 | 6710948 | G | A | C3 | intronic | . | rs10411506 |
| chr19 | 6679360 | T | C | C3 | intronic | . | rs344555 |
| chr19 | 6702455 | G | C | C3 | intronic | . | rs11569450 |
| chr19 | 6678474 | G | A | C3 | splicing | NM_000064:exon40:c.4631-8C>T | rs11569565 |
| chr19 | 6685983 | G | C | C3 | intronic | . | rs2241392 |
| chr19 | 6713291 | C | T | C3 | exonic | . | rs2230201 |
| chr19 | 6709704 | C | T | C3 | exonic | . | rs2230205 |
| chr19 | 6718042 | C | T | C3 | intronic | . | rs11569411 |
| chr19 | 6681991 | G | A | C3 | exonic | . | rs7951 |
| chr19 | 6709848 | C | T | C3 | exonic | . | rs2230204 |
| chr19 | 41808432 | A | T | HNRNPUL1 | intronic | . | rs11881940 |
| chr19 | 10385743 | C | G | ICAM1 | intronic | . | rs5030352 |
| chr19 | 10395973 | G | A | ICAM1 | UTR3 | NM_000201:c.*10G>A | rs2071440 |
| chr19 | 10385540 | A | T | ICAM1 | exonic | . | rs5491 |
| chr19 | 10395248 | C | T | ICAM1 | exonic | . | rs13306429 |
| chr19 | 10395683 | A | G | ICAM1 | exonic | . | rs5498 |
| chr19 | 39789115 | A | G | IFNL1 | exonic | . | rs30461 |
| chr19 | 39760435 | T | C | IFNL2 | exonic | . | rs59746524 |
| chr19 | 39759918 | T | G | IFNL2 | intronic | . | rs62120533 |
| chr19 | 39760336 | T | G | IFNL2 | intronic | . | rs58736064 |
| chr19 | 39759974 | T | C | IFNL2 | intronic | . | rs117789288 |
| chr19 | 39734923 | A | G | IFNL3 | intronic | . | rs11881222 |
| chr19 | 39735106 | T | C | IFNL3 | exonic | . | rs8103142 |
| chr19 | 39734325 | G | A | IFNL3 | exonic | . | rs150748693 |
| chr19 | 39737866 | C | G | IFNL4 | exonic | . | rs12971396 |
| chr19 | 39739129 | C | G | IFNL4 | exonic | . | rs4803221 |
| chr19 | 39738787 | C | T | IFNL4 | intronic | . | rs12979860 |
| chr19 | 39739155 | T | G | IFNL4 | splicing | NM_001276254:exon2:c.64+1A>C,NM_001276254:exon3:c.65-2A>C | rs74597329 |
| chr19 | 50162909 | C | G | IRF3 | exonic | . | rs7251 |
| chr19 | 44084351 | G | T | PINLYP | intronic | . | rs17655484 |
| chr19 | 44084672 | G | C | PINLYP | intronic | . | rs11671498 |
| chr19 | 41854052 | C | A | TGFB1 | intronic | . | rs2241717 |
| chr19 | 41854086 | C | T | TGFB1 | intronic | . | rs2241716 |
| chr19 | 41858921 | G | A | TGFB1 | exonic | . | rs1800470 |
| chr19 | 6669934 | G | A | TNFSF14 | exonic | . | rs2291668 |
| chr19 | 6670253 | A | C | TNFSF14 | intronic | . | rs344558 |
| chr19 | 6665481 | A | G | TNFSF14 | intronic | . | rs8101047 |
| chr19 | 44065388 | C | T | XRCC1 | intronic | . | rs1001581 |
| chr19 | 44049861 | G | T | XRCC1 | intronic | . | rs25478 |
| chr19 | 44050195 | C | T | XRCC1 | splicing | NM_006297:exon14:c.1481+9G>A | rs25479 |
| chr19 | 44047550 | T | C | XRCC1 | exonic | . | rs3547 |
| chr19 | 44057972 | G | A | XRCC1 | intronic | . | rs731420 |
| chr19 | 44056788 | G | A | XRCC1 | intronic | . | rs3213363 |
| chr19 | 44056052 | A | G | XRCC1 | intronic | . | rs25485 |
| chr19 | 44055726 | T | C | XRCC1 | exonic | . | rs25487 |
| chr19 | 44057574 | G | A | XRCC1 | exonic | . | rs1799782 |
| chr19 | 44057227 | T | C | XRCC1 | exonic | . | rs915927 |
| chr19 | 44055644 | G | A | XRCC1 | intronic | . | rs3213368 |
| chr19 | 44055898 | C | T | XRCC1 | intronic | . | rs25486 |
| chr19 | 44057276 | G | A | XRCC1 | intronic | . | rs1799780 |
| chr19 | 44056412 | C | T | XRCC1 | exonic | . | rs25489 |
| chr19 | 44079687 | G | A | XRCC1 | UTR5 | NM_006297:c.-77C>T | rs3213245 |
| chr19 | 53889551 | C | G | ZNF525 | ncRNA_exonic | . | rs4595898 |
| chr2 | 204594512 | T | C | CD28 | intronic | . | rs3116496 |
| chr2 | 204732714 | A | G | CTLA4 | exonic | . | rs231775 |
| chr2 | 219029108 | C | G | CXCR1 | exonic | . | rs2234671 |
| chr2 | 219000310 | C | T | CXCR2 | exonic | . | rs2230054 |
| chr2 | 218925149 | G | A | CXCR2P1 | ncRNA_exonic | . | rs6758271 |
| chr2 | 113540177 | C | T | IL1A | intronic | . | rs1894399 |
| chr2 | 113540205 | G | T | IL1A | intronic | . | rs1609682 |
| chr2 | 113537352 | T | G | IL1A | intronic | . | rs2071374 |
| chr2 | 113532885 | G | T | IL1A | intronic | . | rs3783550 |
| chr2 | 113535395 | G | T | IL1A | intronic | . | rs2071376 |
| chr2 | 113535438 | C | T | IL1A | intronic | . | rs2071375 |
| chr2 | 113537223 | C | A | IL1A | exonic | . | rs17561 |
| chr2 | 113537339 | A | G | IL1A | intronic | . | rs2856841 |
| chr2 | 113590467 | C | T | IL1B | intronic | . | rs1143633 |
| chr2 | 113588302 | C | T | IL1B | intronic | . | rs1143643 |
| chr2 | 113591275 | A | G | IL1B | intronic | . | rs3136558 |
| chr2 | 227662189 | A | G | IRS1 | exonic | . | rs3731596 |
| chr2 | 227661043 | T | C | IRS1 | exonic | . | rs1801123 |
| chr2 | 227659583 | G | C | IRS1 | intronic | . | rs3769647 |
| chr2 | 227660046 | C | T | IRS1 | exonic | . | rs3731594 |
| chr2 | 242795312 | C | G | PDCD1 | intronic | . | rs7419333 |
| chr2 | 242800900 | G | A | PDCD1 | intronic | . | rs35933396 |
| chr2 | 242795350 | A | G | PDCD1 | intronic | . | rs7421861 |
| chr2 | 242800789 | C | G | PDCD1 | intronic | . | rs56211622 |
| chr2 | 242794620 | C | T | PDCD1 | intronic | . | rs34819629 |
| chr2 | 242793433 | G | A | PDCD1 | exonic | . | rs2227982 |
| chr2 | 242793273 | A | G | PDCD1 | exonic | . | rs2227981 |
| chr2 | 242793559 | T | C | PDCD1 | intronic | . | rs6705653 |
| chr2 | 234669144 | G | A | UGT1A1 | exonic | . | rs4148323 |
| chr2 | 234545861 | C | T | UGT1A10 | exonic | . | rs17854828 |
| chr2 | 234637912 | T | C | UGT1A3 | exonic | . | rs6431625 |
| chr2 | 234637803 | T | C | UGT1A3 | exonic | . | rs3821242 |
| chr2 | 234637853 | G | A | UGT1A3 | exonic | . | rs6706232 |
| chr2 | 234638249 | A | G | UGT1A3 | exonic | . | rs7574296 |
| chr2 | 234627608 | T | G | UGT1A4 | exonic | . | rs2011425 |
| chr2 | 234627914 | T | C | UGT1A4 | exonic | . | rs12468274 |
| chr2 | 234628270 | G | A | UGT1A4 | exonic | . | rs3732217 |
| chr2 | 234622429 | T | C | UGT1A5 | exonic | . | rs2012734 |
| chr2 | 234622110 | C | G | UGT1A5 | exonic | . | rs12475068 |
| chr2 | 234622310 | C | T | UGT1A5 | exonic | . | rs17862867 |
| chr2 | 234622382 | G | C | UGT1A5 | exonic | . | rs17862868 |
| chr2 | 234622420 | T | C | UGT1A5 | exonic | . | rs17862869 |
| chr2 | 234622294 | C | T | UGT1A5 | exonic | . | rs17863790 |
| chr2 | 234622282 | C | T | UGT1A5 | exonic | . | rs17868333 |
| chr2 | 234622379 | C | A | UGT1A5 | exonic | . | rs2012736 |
| chr2 | 234622061 | C | A | UGT1A5 | exonic | . | rs3755320 |
| chr2 | 234621825 | T | C | UGT1A5 | exonic | . | rs3755321 |
| chr2 | 234621787 | C | G | UGT1A5 | exonic | . | rs3755322 |
| chr2 | 234621780 | T | C | UGT1A5 | exonic | . | rs3755323 |
| chr2 | 234622412 | G | C | UGT1A5 | exonic | . | rs3892170 |
| chr2 | 234638690 | A | T | UGT1A5,UGT1A3,UGT1A8,UGT1A10,UGT1A6,UGT1A9,UGT1A7,UGT1A4 | intronic | . | rs2361501 |
| chr2 | 234656500 | G | C | UGT1A5,UGT1A3,UGT1A8,UGT1A10,UGT1A6,UGT1A9,UGT1A7,UGT1A4 | intronic | . | rs28900382 |
| chr2 | 234656640 | T | C | UGT1A5,UGT1A3,UGT1A8,UGT1A10,UGT1A6,UGT1A9,UGT1A7,UGT1A4 | intronic | . | rs11888459 |
| chr2 | 234668870 | G | C | UGT1A5,UGT1A3,UGT1A8,UGT1A10,UGT1A6,UGT1A9,UGT1A7,UGT1A4 | intronic | . | rs873478 |
| chr2 | 234656517 | T | C | UGT1A5,UGT1A3,UGT1A8,UGT1A10,UGT1A6,UGT1A9,UGT1A7,UGT1A4 | intronic | . | rs12466997 |
| chr2 | 234656735 | C | T | UGT1A5,UGT1A3,UGT1A8,UGT1A10,UGT1A6,UGT1A9,UGT1A7,UGT1A4 | intronic | . | rs17863798 |
| chr2 | 234675826 | T | C | UGT1A5,UGT1A8,UGT1A10,UGT1A6,UGT1A7,UGT1A3,UGT1A1,UGT1A9,UGT1A4 | intronic | . | rs4148327 |
| chr2 | 234627304 | G | A | UGT1A5,UGT1A8,UGT1A10,UGT1A6,UGT1A9,UGT1A7 | intronic | . | rs3732218 |
| chr2 | 234637707 | T | C | UGT1A5,UGT1A8,UGT1A10,UGT1A6,UGT1A9,UGT1A7,UGT1A4 | intronic | . | rs3806596 |
| chr2 | 234637569 | A | G | UGT1A5,UGT1A8,UGT1A10,UGT1A6,UGT1A9,UGT1A7,UGT1A4 | intronic | . | rs3806597 |
| chr2 | 234628529 | T | C | UGT1A5,UGT1A8,UGT1A10,UGT1A6,UGT1A9,UGT1A7,UGT1A4 | intronic | . | rs871514 |
| chr2 | 234628376 | C | T | UGT1A5,UGT1A8,UGT1A10,UGT1A6,UGT1A9,UGT1A7,UGT1A4 | intronic | . | rs2011219 |
| chr2 | 234591339 | G | A | UGT1A7 | exonic | . | rs17864686 |
| chr2 | 234590974 | C | A | UGT1A7 | exonic | . | rs17863778 |
| chr2 | 234590970 | T | G | UGT1A7 | exonic | . | rs17868323 |
| chr2 | 234590975 | G | A | UGT1A7 | exonic | . | rs17868324 |
| chr2 | 234591205 | T | C | UGT1A7 | exonic | . | rs11692021 |
| chr2 | 234590616 | C | A | UGT1A7 | exonic | . | rs7577677 |
| chr2 | 234527118 | A | G | UGT1A8 | exonic | . | rs1042605 |
| chr2 | 234526871 | C | G | UGT1A8 | exonic | . | rs1042597 |
| chr2 | 234557478 | C | T | UGT1A8,UGT1A10 | intronic | . | rs193192609 |
| chr2 | 234556711 | C | A | UGT1A8,UGT1A10 | intronic | . | rs145242971 |
| chr2 | 234546157 | G | C | UGT1A8,UGT1A10 | intronic | . | rs1901814 |
| chr2 | 234557414 | T | C | UGT1A8,UGT1A10 | intronic | . | rs28444828 |
| chr2 | 234621404 | C | T | UGT1A8,UGT1A10,UGT1A6,UGT1A9,UGT1A7 | intronic | . | rs4556969 |
| chr2 | 234581587 | G | A | UGT1A8,UGT1A10,UGT1A9 | intronic | . | rs4663871 |
| chr2 | 234581654 | A | T | UGT1A8,UGT1A10,UGT1A9 | intronic | . | rs2741047 |
| chr2 | 234590527 | T | G | UGT1A8,UGT1A10,UGT1A9 | intronic | . | rs7586110 |
| chr2 | 216982370 | C | G | XRCC5 | intronic | . | rs3815855 |
| chr2 | 217059809 | G | A | XRCC5 | intronic | . | rs3821102 |
| chr2 | 217069151 | A | G | XRCC5 | intronic | . | rs1438162 |
| chr20 | 60058231 | A | G | CDH4 | intronic | . | rs1000322 |
| chr20 | 44645153 | T | C | MMP9 | UTR3 | NM_004994:c.*146T>C | rs9509 |
| chr20 | 44642833 | A | C | MMP9 | exonic | . | rs13969 |
| chr20 | 44640391 | C | T | MMP9 | splicing | NM_004994:exon6:c.997+5C>T | rs3918254 |
| chr20 | 44640229 | C | T | MMP9 | exonic | . | rs45437897 |
| chr20 | 44641731 | G | A | MMP9 | intronic | . | rs3787268 |
| chr20 | 44640225 | A | G | MMP9 | exonic | . | rs17576 |
| chr20 | 44640575 | A | G | MMP9 | intronic | . | rs2236416 |
| chr20 | 44639692 | G | T | MMP9 | splicing | NM_004994:exon4:c.649+3G>T | rs2274755 |
| chr20 | 44644965 | G | A | MMP9 | exonic | . | rs13925 |
| chr20 | 44643111 | G | A | MMP9 | exonic | . | rs17577 |
| chr20 | 44642406 | G | C | MMP9 | exonic | . | rs2250889 |
| chr20 | 44638781 | A | G | MMP9 | intronic | . | rs3918251 |
| chr20 | 50015299 | A | C | NFATC2 | intronic | . | rs2426295 |
| chr20 | 50179356 | C | T | NFATC2 | UTR5 | NM_001136021:c.-188G>A;NM_001258292:c.-188G>A;NM_001258294:c.-39234G>A;NM_001258295:c.-39234G>A | rs75374025 |
| chr20 | 50091937 | A | G | NFATC2 | intronic | . | rs56332276 |
| chr20 | 50071258 | A | G | NFATC2 | intronic | . | rs228840 |
| chr20 | 50092027 | T | G | NFATC2 | exonic | . | rs6013193 |
| chr20 | 50092287 | T | C | NFATC2 | intronic | . | rs6021231 |
| chr20 | 50178965 | G | C | NFATC2 | intronic | . | rs2869427 |
| chr20 | 52492074 | G | A | SUMO1P1 | ncRNA_exonic | . | rs6068699 |
| chr20 | 52491761 | T | C | SUMO1P1 | ncRNA_exonic | . | rs11699573 |
| chr21 | 36165041 | T | C | RUNX1 | intronic | . | rs11700756 |
| chr21 | 36421036 | T | C | RUNX1 | intronic | . | rs8133634 |
| chr21 | 36164486 | G | C | RUNX1 | exonic | . | rs61750222 |
| chr21 | 46228170 | T | G | SUMO3 | intronic | . | rs188978703 |
| chr21 | 46234079 | T | A | SUMO3 | intronic | . | rs9306116 |
| chr21 | 46233836 | C | A | SUMO3 | exonic | . | rs2838697 |
| chr21 | 46233863 | G | C | SUMO3 | exonic | . | rs13050872 |
| chr21 | 46228165 | T | C | SUMO3 | intronic | . | rs7283639 |
| chr22 | 19950428 | A | G | COMT | intronic | . | rs2239393 |
| chr22 | 19951207 | C | G | COMT | exonic | . | rs4818 |
| chr22 | 19950235 | C | T | COMT | exonic | . | rs4633 |
| chr22 | 19949952 | A | G | COMT | intronic | . | rs6269 |
| chr22 | 19954847 | G | A | COMT | intronic | . | rs4646318 |
| chr22 | 19951271 | G | A | COMT | exonic | . | rs4680 |
| chr22 | 19951804 | G | A | COMT | exonic | . | rs769224 |
| chr22 | 19951897 | G | C | COMT | intronic | . | rs4646315 |
| chr22 | 37528606 | G | A | IL2RB | intronic | . | rs2072862 |
| chr22 | 37539713 | T | C | IL2RB | intronic | . | rs2235330 |
| chr22 | 37533786 | C | G | IL2RB | intronic | . | rs228957 |
| chr22 | 37533795 | G | C | IL2RB | intronic | . | rs228958 |
| chr22 | 37531521 | G | C | IL2RB | intronic | . | rs3218305 |
| chr22 | 37533530 | G | C | IL2RB | intronic | . | rs3218292 |
| chr22 | 37532514 | C | T | IL2RB | intronic | . | rs3218296 |
| chr22 | 37532441 | G | A | IL2RB | splicing | NM_000878:exon8:c.538-8C>T | rs3218297 |
| chr22 | 37532207 | C | T | IL2RB | intronic | . | rs3218299 |
| chr22 | 37535328 | C | G | IL2RB | intronic | . | rs2281093 |
| chr22 | 37532172 | A | G | IL2RB | intronic | . | rs2281089 |
| chr22 | 37524619 | G | T | IL2RB | exonic | . | rs228942 |
| chr22 | 37531436 | G | A | IL2RB | exonic | . | rs228953 |
| chr22 | 37528576 | A | G | IL2RB | intronic | . | rs3218318 |
| chr22 | 37528362 | T | G | IL2RB | intronic | . | rs2072861 |
| chr22 | 22127022 | T | C | MAPK1 | intronic | . | rs142981654 |
| chr22 | 22153239 | G | A | MAPK1 | intronic | . | rs2298434 |
| chr22 | 50693521 | C | T | MAPK12 | intronic | . | rs5771282 |
| chr22 | 50693534 | A | G | MAPK12 | intronic | . | rs6010218 |
| chr22 | 50699668 | A | G | MAPK12 | exonic | . | rs2272857 |
| chr22 | 50696662 | C | T | MAPK12 | splicing | NM_001303252:exon4:c.314+10G>A;NM_002969:exon4:c.314+10G>A | rs73187284 |
| chr22 | 50694297 | A | G | MAPK12 | exonic | . | rs1129880 |
| chr22 | 50693479 | A | G | MAPK12 | intronic | . | rs5771281 |
| chr22 | 50695270 | A | C | MAPK12 | intronic | . | rs5771284 |
| chr22 | 50693538 | G | A | MAPK12 | intronic | . | rs6010219 |
| chr22 | 50693554 | A | G | MAPK12 | intronic | . | rs6010220 |
| chr22 | 50693889 | G | A | MAPK12 | exonic | . | rs2066770 |
| chr22 | 50696648 | C | T | MAPK12 | intronic | . | rs2235355 |
| chr22 | 50695370 | G | A | MAPK12 | exonic | . | rs2072876 |
| chr22 | 50693558 | C | T | MAPK12 | intronic | . | rs74798979 |
| chr22 | 50696678 | G | A | MAPK12 | exonic | . | rs34422484 |
| chr22 | 46614469 | G | A | PPARA | intronic | . | rs5767708 |
| chr22 | 42059768 | G | T | XRCC6 | exonic | . | rs132788 |
| chr3 | 148459395 | C | T | AGTR1 | exonic | . | rs5182 |
| chr3 | 148457642 | T | C | AGTR1 | UTR5 | NM_032049:c.-62T>C | rs1800766 |
| chr3 | 112185025 | G | A | BTLA | exonic | . | rs9288952 |
| chr3 | 112184927 | A | G | BTLA | UTR3 | NM_001085357:c.*28T>C;NM_181780:c.*28T>C | rs2171513 |
| chr3 | 112188609 | T | G | BTLA | exonic | . | rs76844316 |
| chr3 | 119263680 | C | T | CD80 | exonic | . | rs2228017 |
| chr3 | 121838319 | G | A | CD86 | exonic | . | rs1129055 |
| chr3 | 33118951 | A | G | GLB1 | intronic | . | rs9827863 |
| chr3 | 49064110 | A | G | IMPDH2 | splicing | NM_000884:exon8:c.819+10T>C | rs11706052 |
| chr3 | 119533733 | G | A | NR1I2 | intronic | . | rs6785049 |
| chr3 | 119534153 | C | T | NR1I2 | intronic | . | rs2276707 |
| chr3 | 119501506 | T | G | NR1I2 | intronic | . | rs3814056 |
| chr3 | 119526349 | G | A | NR1I2 | intronic | . | rs1464603 |
| chr3 | 119501965 | G | A | NR1I2 | intronic | . | rs74825699 |
| chr3 | 119526372 | G | A | NR1I2 | intronic | . | rs1464602 |
| chr3 | 12475557 | C | T | PPARG | exonic | . | rs3856806 |
| chr3 | 51707875 | G | A | TEX264 | intronic | . | rs76735914 |
| chr3 | 30686414 | A | G | TGFBR2 | splicing | NM_001024847:exon3:c.338+7A>G;NM_003242:exon2:c.263+7A>G | rs1155705 |
| chr3 | 30732821 | C | A | TGFBR2 | intronic | . | rs2276767 |
| chr3 | 30713126 | T | A | TGFBR2 | splicing | NM_001024847:exon5:c.530-4T>A;NM_003242:exon4:c.455-4T>A | rs11466512 |
| chr3 | 30713945 | T | C | TGFBR2 | intronic | . | rs45515293 |
| chr3 | 30713842 | C | T | TGFBR2 | exonic | . | rs2228048 |
| chr3 | 52258372 | T | C | TLR9 | intronic | . | rs352139 |
| chr3 | 52256697 | C | T | TLR9 | exonic | . | rs352140 |
| chr4 | 76944785 | C | T | ART3 | intronic | . | rs56061981 |
| chr4 | 76956179 | G | A | ART3,CXCL11 | intronic | . | rs12649185 |
| chr4 | 76957043 | G | A | ART3,CXCL11 | intronic | . | rs6817952 |
| chr4 | 76928428 | C | T | CXCL9 | intronic | . | rs2276886 |
| chr4 | 148461037 | T | C | EDNRA | exonic | . | rs5333 |
| chr4 | 148461073 | G | A | EDNRA | exonic | . | rs5334 |
| chr4 | 148460831 | G | A | EDNRA | intronic | . | rs6840375 |
| chr4 | 148463840 | G | C | EDNRA | UTR3 | NM_001166055:c.*70G>C;NM_001256283:c.*70G>C;NM_001957:c.*70G>C | rs5335 |
| chr4 | 148441139 | G | A | EDNRA | splicing | NM_001957:exon3:c.548+9G>A | rs4835412 |
| chr4 | 123377482 | C | A | IL2 | exonic | . | rs2069763 |
| chr4 | 123536963 | G | A | IL21 | exonic | . | rs4833837 |
| chr4 | 103514741 | T | C | NFKB1 | intronic | . | rs4648050 |
| chr4 | 103534560 | C | G | NFKB1 | intronic | . | rs3817685 |
| chr4 | 103537442 | C | T | NFKB1 | intronic | . | rs1609798 |
| chr4 | 103533821 | T | A | NFKB1 | intronic | . | rs4648110 |
| chr4 | 82135230 | G | A | PRKG2 | intronic | . | rs710840 |
| chr4 | 77589959 | A | G | SHROOM3 | intronic | . | rs7691621 |
| chr4 | 9892102 | T | C | SLC2A9 | intronic | . | rs10939602 |
| chr4 | 9987324 | G | A | SLC2A9 | exonic | . | rs3733589 |
| chr4 | 9987226 | T | C | SLC2A9 | intronic | . | rs3733590 |
| chr4 | 10020480 | C | T | SLC2A9 | intronic | . | rs2240720 |
| chr4 | 9909923 | G | A | SLC2A9 | exonic | . | rs2280205 |
| chr4 | 9836366 | T | C | SLC2A9 | intronic | . | rs3775950 |
| chr4 | 9998440 | C | T | SLC2A9 | exonic | . | rs10939650 |
| chr4 | 10022880 | T | C | SLC2A9 | intronic | . | rs2276962 |
| chr4 | 9982191 | C | T | SLC2A9 | intronic | . | rs13115193 |
| chr4 | 10027354 | C | G | SLC2A9 | intronic | . | rs6819833 |
| chr4 | 10027643 | A | G | SLC2A9 | intronic | . | rs6449237 |
| chr4 | 10027744 | G | A | SLC2A9 | intronic | . | rs6449238 |
| chr4 | 10027542 | C | T | SLC2A9 | exonic | . | rs6820230 |
| chr4 | 10022839 | G | A | SLC2A9 | intronic | . | rs2276963 |
| chr4 | 9922130 | C | T | SLC2A9 | exonic | . | rs3733591 |
| chr4 | 9909850 | T | G | SLC2A9 | splicing | NM_001001290:exon10:c.1026+9A>C;NM_020041:exon9:c.1113+9A>C | rs2280204 |
| chr4 | 9943700 | G | A | SLC2A9 | intronic | . | rs4292327 |
| chr4 | 10020564 | G | A | SLC2A9 | intronic | . | rs2240721 |
| chr4 | 9921931 | T | C | SLC2A9 | intronic | . | rs6823877 |
| chr4 | 10020757 | A | G | SLC2A9 | intronic | . | rs2240722 |
| chr4 | 10022981 | C | T | SLC2A9 | exonic | . | rs2276961 |
| chr4 | 9889069 | C | T | SLC2A9 | intronic | . | rs6836878 |
| chr4 | 88904186 | A | G | SPP1 | UTR3 | NM_000582:c.*138A>G;NM_001040058:c.*138A>G;NM_001040060:c.*138A>G;NM_001251829:c.*138A>G;NM_001251830:c.*138A>G | rs1126772 |
| chr4 | 88903853 | C | T | SPP1 | exonic | . | rs1126616 |
| chr4 | 88902692 | T | C | SPP1 | exonic | . | rs4754 |
| chr4 | 88898941 | C | T | SPP1 | exonic | . | rs11728697 |
| chr4 | 154624656 | T | C | TLR2 | exonic | . | rs3804099 |
| chr4 | 154625409 | T | C | TLR2 | exonic | . | rs3804100 |
| chr4 | 70465153 | A | T | UGT2A2,UGT2A1 | intronic | . | rs3775782 |
| chr4 | 69879718 | A | G | UGT2B10 | intronic | . | rs2292524 |
| chr4 | 69875627 | G | A | UGT2B10 | splicing | NM_001075:exon7:c.1056+7C>T;NM_001144767:exon7:c.804+7C>T;NM_001290091:exon6:c.343+7C>T | rs2331627 |
| chr4 | 69870721 | A | G | UGT2B10 | exonic | . | rs111542944 |
| chr4 | 70079963 | G | A | UGT2B11 | exonic | . | rs72551399 |
| chr4 | 70078507 | A | G | UGT2B11 | intronic | . | rs62298956 |
| chr4 | 70070366 | A | T | UGT2B11 | exonic | . | rs144106768 |
| chr4 | 70080093 | C | T | UGT2B11 | exonic | . | rs150042325 |
| chr4 | 70066319 | G | T | UGT2B11 | exonic | . | rs72551394 |
| chr4 | 70079838 | T | C | UGT2B11 | exonic | . | rs4694697 |
| chr4 | 70160309 | C | G | UGT2B28 | exonic | . | rs6828191 |
| chr4 | 70160277 | T | G | UGT2B28 | exonic | . | rs6843900 |
| chr4 | 70146704 | G | A | UGT2B28 | exonic | . | rs7689398 |
| chr4 | 70156392 | A | G | UGT2B28 | exonic | . | rs10013145 |
| chr4 | 70160338 | G | C | UGT2B28 | exonic | . | rs72552703 |
| chr4 | 70156313 | T | A | UGT2B28 | exonic | . | rs4235127 |
| chr4 | 70352266 | A | G | UGT2B4 | intronic | . | rs1826690 |
| chr4 | 69972949 | C | G | UGT2B7 | exonic | . | rs4292394 |
| chr4 | 69964337 | A | T | UGT2B7 | exonic | . | rs7438284 |
| chr4 | 69964338 | T | C | UGT2B7 | exonic | . | rs7439366 |
| chr4 | 69964209 | A | G | UGT2B7 | intronic | . | rs7438244 |
| chr4 | 69964180 | T | C | UGT2B7 | intronic | . | rs7439326 |
| chr4 | 69962610 | A | G | UGT2B7 | exonic | . | rs28365063 |
| chr4 | 69962449 | G | T | UGT2B7 | exonic | . | rs12233719 |
| chr4 | 69973044 | A | T | UGT2B7 | intronic | . | rs4337789 |
| chr5 | 135276814 | G | A | FBXL21 | exonic | . | rs2066913 |
| chr5 | 135276847 | T | C | FBXL21 | exonic | . | rs31547 |
| chr5 | 135276701 | T | C | FBXL21 | intronic | . | rs31548 |
| chr5 | 135276314 | C | T | FBXL21 | exonic | . | rs40986 |
| chr5 | 132437531 | C | T | HSPA4 | exonic | . | rs1131809 |
| chr5 | 132412299 | A | G | HSPA4 | intronic | . | rs11747001 |
| chr5 | 132409074 | A | G | HSPA4 | intronic | . | rs56121115 |
| chr5 | 132409650 | C | T | HSPA4 | intronic | . | rs62375234 |
| chr5 | 132409867 | T | G | HSPA4 | intronic | . | rs62375235 |
| chr5 | 132388068 | T | G | HSPA4 | intronic | . | rs76836760 |
| chr5 | 132435488 | C | T | HSPA4 | intronic | . | rs4301229 |
| chr5 | 132439820 | C | G | HSPA4 | intronic | . | rs68121884 |
| chr5 | 132424743 | C | T | HSPA4 | splicing | NM_002154:exon10:c.1138-4C>T | rs72667113 |
| chr5 | 132425169 | G | A | HSPA4 | intronic | . | rs6871331 |
| chr5 | 132403341 | C | T | HSPA4 | intronic | . | rs6596098 |
| chr5 | 132425465 | A | G | HSPA4 | intronic | . | rs10076134 |
| chr5 | 132009710 | C | T | IL4 | UTR5 | NM_000589:c.-33C>T;NM_172348:c.-33C>T | rs2070874 |
| chr5 | 132018132 | A | G | IL4 | intronic | . | rs2243289 |
| chr5 | 132018169 | C | A | IL4 | splicing | NM_000589:exon4:c.361-9C>A;NM_172348:exon3:c.313-9C>A | rs2243290 |
| chr5 | 142693846 | A | G | NR3C1 | intronic | . | rs4986593 |
| chr5 | 142662280 | G | A | NR3C1 | exonic | . | rs258751 |
| chr5 | 142678361 | G | A | NR3C1 | exonic | . | rs6194 |
| chr5 | 142661490 | A | G | NR3C1 | exonic | . | rs6196 |
| chr5 | 142680344 | C | A | NR3C1 | intronic | . | rs6188 |
| chr5 | 150037798 | C | T | SYNPO | UTR3 | NM_007286:c.*1149C>T | rs28298 |
| chr5 | 82499307 | G | A | XRCC4 | intronic | . | rs2662238 |
| chr5 | 82649162 | T | C | XRCC4 | UTR3 | NM_003401:c.*101T>C;NM_022406:c.*101T>C;NM_022550:c.*101T>C | rs28360342 |
| chr5 | 82407098 | G | A | XRCC4 | intronic | . | rs1478482 |
| chr5 | 82500734 | G | T | XRCC4 | exonic | . | rs3734091 |
| chr5 | 82648943 | G | A | XRCC4 | splicing | NM_003401:exon8:c.894-7G>A;NM_022406:exon8:c.894-1G>A;NM_022550:exon8:c.894-7G>A | rs1805377 |
| chr5 | 82648977 | T | G | XRCC4 | exonic | . | rs1056503 |
| chr5 | 82648883 | A | G | XRCC4 | intronic | . | rs3777015 |
| chr5 | 82407105 | A | G | XRCC4 | intronic | . | rs1478481 |
| chr6 | 20758943 | G | T | CDKAL1 | intronic | . | rs2820001 |
| chr6 | 20758760 | A | G | CDKAL1 | intronic | . | rs10946406 |
| chr6 | 20740019 | C | T | CDKAL1 | intronic | . | rs4710943 |
| chr6 | 20739907 | C | T | CDKAL1 | intronic | . | rs4710942 |
| chr6 | 21231034 | C | G | CDKAL1 | intronic | . | rs12200511 |
| chr6 | 20781290 | G | A | CDKAL1 | intronic | . | rs12199265 |
| chr6 | 21231007 | G | A | CDKAL1 | intronic | . | rs9460612 |
| chr6 | 21201493 | G | A | CDKAL1 | exonic | . | rs9465994 |
| chr6 | 21065450 | A | G | CDKAL1 | exonic | . | rs56087852 |
| chr6 | 21065449 | C | T | CDKAL1 | exonic | . | rs77152992 |
| chr6 | 20546826 | C | T | CDKAL1 | intronic | . | rs45559831 |
| chr6 | 12292772 | G | T | EDN1 | intronic | . | rs2070699 |
| chr6 | 12296255 | G | T | EDN1 | exonic | . | rs5370 |
| chr6 | 12294137 | T | C | EDN1 | intronic | . | rs1800543 |
| chr6 | 108939083 | C | G | FOXO3 | intronic | . | rs12202234 |
| chr6 | 52051274 | G | A | IL17A | intronic | . | rs3819025 |
| chr6 | 52051382 | A | G | IL17A | intronic | . | rs8193038 |
| chr6 | 39306757 | C | T | KIF6 | intronic | . | rs56079646 |
| chr6 | 31540784 | C | A | LTA | exonic | . | rs1041981 |
| chr6 | 36041431 | A | G | MAPK14 | intronic | . | rs851010 |
| chr6 | 36075219 | A | C | MAPK14 | intronic | . | rs9470219 |
| chr6 | 36070227 | G | T | MAPK14 | intronic | . | rs16884857 |
| chr6 | 36041707 | G | T | MAPK14 | intronic | . | rs3730326 |
| chr6 | 149721965 | T | C | SUMO4 | UTR3 | NM_001002255:c.*150T>C | rs237024 |
| chr6 | 149721690 | G | A | SUMO4 | exonic | . | rs237025 |
| chr6 | 43748545 | G | A | VEGFA | exonic;splicing | NM_001171624:exon6:c.494+5G>A;NM_001204384:exon6:c.494+5G>A;NM_001204385:exon6:c.1034+5G>A;NM_003376:exon6:c.1034+5G>A | rs185218985 |
| chr6 | 43745095 | C | A | VEGFA | intronic | . | rs2146323 |
| chr6 | 43748795 | C | T | VEGFA | intronic | . | rs3025018 |
| chr6 | 43745107 | G | A | VEGFA | intronic | . | rs3024997 |
| chr6 | 43752536 | C | T | VEGFA | UTR3 | NM_001025366:c.*237C>T;NM_001025367:c.*237C>T;NM_001025368:c.*237C>T;NM_001025369:c.*253C>T;NM_001025370:c.*237C>T;NM_001033756:c.*171C>T;NM_001171622:c.*237C>T;NM_001171623:c.*237C>T;NM_001171624:c.*237C>T;NM_001171625:c.*237C>T;NM_001171626:c.*237C>T;NM_001171627:c.*253C>T;NM_001171628:c.*237C>T;NM_001171629:c.*171C>T;NM_001171630:c.*237C>T;NM_001204384:c.*237C>T;NM_001204385:c.*237C>T;NM_001287044:c.*237C>T;NM_003376:c.*237C>T | rs3025039 |
| chr6 | 43745577 | C | T | VEGFA | intronic | . | rs3024998 |
| chr6 | 43746169 | C | T | VEGFA | intronic | . | rs3025000 |
| chr6 | 43748357 | G | A | VEGFA | intronic | . | rs3025017 |
| chr6 | 43738350 | C | G | VEGFA | UTR5 | NM_001025366:c.-94C>G;NM_001025367:c.-94C>G;NM_001025368:c.-94C>G;NM_001025369:c.-94C>G;NM_001025370:c.-94C>G;NM_001033756:c.-94C>G;NM_001171622:c.-94C>G;NM_001171623:c.-634C>G;NM_001171624:c.-634C>G;NM_001171625:c.-634C>G;NM_001171626:c.-634C>G;NM_001171627:c.-634C>G;NM_001171628:c.-634C>G;NM_001171629:c.-634C>G;NM_001171630:c.-634C>G;NM_001204384:c.-634C>G;NM_001204385:c.-94C>G;NM_003376:c.-94C>G | rs2010963 |
| chr7 | 87168455 | C | T | ABCB1 | intronic | . | rs2235066 |
| chr7 | 87179086 | G | A | ABCB1 | intronic | . | rs2235035 |
| chr7 | 87174066 | T | C | ABCB1 | intronic | . | rs2235046 |
| chr7 | 87178626 | C | T | ABCB1 | intronic | . | rs2235013 |
| chr7 | 87179143 | A | G | ABCB1 | intronic | . | rs2235033 |
| chr7 | 87183354 | T | C | ABCB1 | intronic | . | rs1922240 |
| chr7 | 87138532 | A | C | ABCB1 | intronic | . | rs2235047 |
| chr7 | 87180198 | C | T | ABCB1 | intronic | . | rs10276036 |
| chr7 | 87179601 | A | G | ABCB1 | exonic | . | rs1128503 |
| chr7 | 87144413 | G | C | ABCB1 | intronic | . | rs4148749 |
| chr7 | 87225046 | G | A | ABCB1 | intronic | . | rs2235074 |
| chr7 | 87138645 | A | G | ABCB1 | exonic | . | rs1045642 |
| chr7 | 87138511 | G | A | ABCB1 | intronic | . | rs2235048 |
| chr7 | 87144816 | G | C | ABCB1 | intronic | . | rs7779562 |
| chr7 | 87160618 | A | C | ABCB1 | exonic | . | rs2032582 |
| chr7 | 150709571 | G | T | ATG9B | UTR3 | NM_173681:c.*1452C>A | rs7830 |
| chr7 | 116166938 | C | T | CAV1 | intronic | . | rs78358369 |
| chr7 | 116165360 | A | C | CAV1 | UTR5 | NM_001172895:c.-1282A>C | rs1997623 |
| chr7 | 116165463 | T | C | CAV1 | UTR5 | NM_001172895:c.-1179T>C | rs2742125 |
| chr7 | 116140524 | G | T | CAV2 | intronic | . | rs2270188 |
| chr7 | 116145957 | A | T | CAV2 | intronic | . | rs4730743 |
| chr7 | 116140275 | T | C | CAV2 | UTR5 | NM_001206748:c.-138T>C | rs11767363 |
| chr7 | 116140149 | G | T | CAV2 | UTR5 | NM_001206748:c.-264G>T | rs13235183 |
| chr7 | 99359483 | T | C | CYP3A4 | intronic | . | rs34382314 |
| chr7 | 99367825 | T | C | CYP3A4 | exonic | . | rs55951658 |
| chr7 | 99365943 | C | A | CYP3A4 | intronic | . | rs2687116 |
| chr7 | 99361466 | C | T | CYP3A4 | intronic | . | rs2242480 |
| chr7 | 99355975 | T | C | CYP3A4 | intronic | . | rs3735451 |
| chr7 | 99459144 | T | C | CYP3A43 | intronic | . | rs503115 |
| chr7 | 99270539 | C | T | CYP3A5 | splicing | . | rs776746 |
| chr7 | 99260362 | C | A | CYP3A5 | intronic | . | rs4646453 |
| chr7 | 99245914 | A | G | CYP3A5 | UTR3 | NM_000777:c.*14T>C;NM_001291829:c.*14T>C;NM_001291830:c.*14T>C | rs15524 |
| chr7 | 99303094 | G | A | CYP3A7 | UTR3 | NM_000765:c.*29C>T | rs12360 |
| chr7 | 99297804 | C | T | CYP3A7-CYP3A51P | intronic | . | rs2177180 |
| chr7 | 99293475 | T | A | CYP3A7-CYP3A51P | splicing | NM_001256497:exon15:c.1498-6A>T | rs2740565 |
| chr7 | 99293358 | A | G | CYP3A7-CYP3A51P | intronic | . | rs72494453 |
| chr7 | 99306685 | C | G | CYP3A7-CYP3A51P,CYP3A7 | exonic | . | rs2257401 |
| chr7 | 99314986 | A | G | CYP3A7-CYP3A51P,CYP3A7 | intronic | . | rs2687075 |
| chr7 | 99306566 | C | T | CYP3A7-CYP3A51P,CYP3A7 | intronic | . | rs2687077 |
| chr7 | 99314727 | A | G | CYP3A7-CYP3A51P,CYP3A7 | intronic | . | rs45600842 |
| chr7 | 22768249 | G | T | IL6 | intronic | . | rs2066992 |
| chr7 | 22768572 | C | G | IL6 | intronic | . | rs2069840 |
| chr7 | 22771296 | G | A | IL6 | UTR3 | NM_000600:c.*104G>A | rs13306436 |
| chr7 | 128034629 | C | T | IMPDH1 | exonic | . | rs2228075 |
| chr7 | 128040752 | C | T | IMPDH1 | intronic | . | rs2278293 |
| chr7 | 128049701 | T | A | IMPDH1 | intronic | . | rs2288553 |
| chr7 | 128049573 | C | T | IMPDH1 | intronic | . | rs2288551 |
| chr7 | 128049590 | C | A | IMPDH1 | intronic | . | rs2288552 |
| chr7 | 128034767 | A | C | IMPDH1 | intronic | . | rs4731447 |
| chr7 | 128040699 | C | T | IMPDH1 | intronic | . | rs2278294 |
| chr7 | 128038555 | C | G | IMPDH1 | exonic | . | rs2288550 |
| chr7 | 128034467 | C | T | IMPDH1 | intronic | . | rs72624969 |
| chr7 | 128041339 | A | G | IMPDH1 | intronic | . | rs4731448 |
| chr7 | 150703915 | G | T | NOS3 | intronic | . | rs2853796 |
| chr7 | 150698879 | A | G | NOS3 | intronic | . | rs1800780 |
| chr7 | 150699250 | G | T | NOS3 | intronic | . | rs1800782 |
| chr7 | 150704400 | C | A | NOS3 | intronic | . | rs3730305 |
| chr7 | 150700637 | A | G | NOS3 | UTR3 | NM_001160110:c.*146A>G;NM_001160111:c.*261A>G | rs73167652 |
| chr7 | 150692444 | G | A | NOS3 | intronic | . | rs1800781 |
| chr7 | 150700291 | G | A | NOS3 | exonic | . | rs79467411 |
| chr7 | 150695726 | T | C | NOS3 | exonic | . | rs1549758 |
| chr7 | 150708850 | G | A | NOS3 | intronic | . | rs2256314 |
| chr7 | 150708089 | A | G | NOS3 | intronic | . | rs891512 |
| chr7 | 150700515 | T | G | NOS3 | UTR3 | NM_001160110:c.*24T>G;NM_001160111:c.*139T>G | rs2566508 |
| chr7 | 150704250 | C | G | NOS3 | exonic | . | rs2566514 |
| chr7 | 150696008 | A | G | NOS3 | intronic | . | rs1007311 |
| chr7 | 150696111 | T | G | NOS3 | exonic | . | rs1799983 |
| chr7 | 150703692 | C | T | NOS3 | intronic | . | rs3729625 |
| chr7 | 75614953 | T | C | POR | exonic | . | rs2228104 |
| chr7 | 75614082 | C | G | POR | intronic | . | rs4732516 |
| chr7 | 55585184 | G | A | VOPP1 | intronic | . | rs11238367 |
| chr8 | 91031074 | G | A | DECR1 | intronic | . | rs1805867 |
| chr8 | 91064036 | G | C | DECR1 | intronic | . | rs1476590 |
| chr8 | 91063787 | G | T | DECR1 | intronic | . | rs7816839 |
| chr8 | 91018507 | T | A | DECR1 | intronic | . | rs16902273 |
| chr8 | 91033080 | A | G | DECR1 | intronic | . | rs74648935 |
| chr8 | 91017745 | T | C | DECR1 | intronic | . | rs1805806 |
| chr8 | 63767814 | T | A | NKAIN3 | intronic | . | rs931129 |
| chr8 | 63769408 | A | T | NKAIN3 | intronic | . | rs10107381 |
| chr8 | 63768397 | A | T | NKAIN3 | intronic | . | rs62510782 |
| chr8 | 63768483 | G | A | NKAIN3 | intronic | . | rs76728266 |
| chr8 | 63769048 | A | C | NKAIN3 | intronic | . | rs78409998 |
| chr8 | 63768015 | T | C | NKAIN3 | intronic | . | rs2017227 |
| chr8 | 63767802 | G | A | NKAIN3 | intronic | . | rs10957248 |
| chr8 | 63768731 | G | A | NKAIN3 | intronic | . | rs899680 |
| chr8 | 48744624 | G | A | PRKDC | intronic | . | rs141915027 |
| chr8 | 48719852 | G | C | PRKDC | exonic | . | rs55793951 |
| chr8 | 48810975 | T | C | PRKDC | intronic | . | rs55960960 |
| chr8 | 48689366 | C | T | PRKDC | intronic | . | rs8178258 |
| chr8 | 48805582 | G | A | PRKDC | intronic | . | rs10097783 |
| chr8 | 48776189 | C | T | PRKDC | intronic | . | rs6993483 |
| chr8 | 48772414 | C | T | PRKDC | intronic | . | rs7839407 |
| chr8 | 48711682 | C | G | PRKDC | intronic | . | rs7828380 |
| chr8 | 48792042 | A | T | PRKDC | splicing | NM_001081640:exon40:c.5234+10T>A;NM_006904:exon40:c.5234+10T>A | rs8178111 |
| chr8 | 48801480 | C | T | PRKDC | intronic | . | rs3213447 |
| chr8 | 48710955 | A | G | PRKDC | exonic | . | rs7830743 |
| chr8 | 48852328 | C | T | PRKDC | intronic | . | rs8178016 |
| chr8 | 48771301 | T | C | PRKDC | intronic | . | rs8178148 |
| chr8 | 118184783 | C | T | SLC30A8 | exonic | . | rs13266634 |
| chr8 | 118185025 | G | A | SLC30A8 | UTR3 | NM_001172811:c.*105G>A;NM_001172813:c.*105G>A;NM_001172814:c.*105G>A;NM_001172815:c.*105G>A;NM_173851:c.*105G>A | rs3802177 |
| chr8 | 118165149 | A | G | SLC30A8 | intronic | . | rs6469675 |
| chr9 | 123785545 | G | A | C5 | intronic | . | rs28426093 |
| chr9 | 123726145 | T | C | C5 | intronic | . | rs2300931 |
| chr9 | 123760173 | C | T | C5 | intronic | . | rs13297393 |
| chr9 | 123725971 | C | T | C5 | exonic | . | rs12237774 |
| chr9 | 123800218 | C | T | C5 | exonic | . | rs17216529 |
| chr9 | 123769200 | C | T | C5 | exonic | . | rs17611 |
| chr9 | 123789634 | C | T | C5 | intronic | . | rs2416811 |
| chr9 | 123760107 | G | C | C5 | intronic | . | rs10985118 |
| chr9 | 123737018 | C | T | C5 | intronic | . | rs2269066 |
| chr9 | 123780005 | G | A | C5 | exonic | . | rs25681 |
| chr9 | 123783934 | T | C | C5 | exonic | . | rs10985126 |
| chr9 | 123783993 | A | G | C5 | intronic | . | rs10985127 |
| chr9 | 123737040 | G | C | C5 | intronic | . | rs2269067 |
| chr9 | 5467955 | G | A | CD274 | UTR3 | NM_001267706:c.*93G>A;NM_014143:c.*93G>A | rs2297136 |
| chr9 | 5465732 | G | A | CD274 | intronic | . | rs2297137 |
| chr9 | 99060660 | A | G | HSD17B3 | intronic | . | rs8190504 |
| chr9 | 98997810 | C | T | HSD17B3 | exonic | . | rs2066479 |
| chr9 | 99064425 | T | C | HSD17B3 | UTR5 | NM_000197:c.-39A>G | rs2066474 |
| chr9 | 99064415 | T | C | HSD17B3 | UTR5 | NM_000197:c.-29A>G | rs2066476 |
| chr9 | 99060612 | T | C | HSD17B3 | intronic | . | rs7029101 |
| chr9 | 99003264 | C | T | HSD17B3 | intronic | . | rs2066485 |
| chr9 | 99013663 | A | G | HSD17B3 | intronic | . | rs408876 |
| chr9 | 99002854 | G | A | HSD17B3 | intronic | . | rs8190557 |
| chr9 | 6253571 | C | T | IL33 | exonic | . | rs10975519 |
| chr9 | 6255881 | T | G | IL33 | intronic | . | rs1332290 |
| chr9 | 6256292 | G | A | IL33 | UTR3 | NM_001199640:c.*124G>A;NM_001199641:c.*124G>A;NM_033439:c.*124G>A | rs1048274 |
| chr9 | 6253710 | G | C | IL33 | intronic | . | rs10975520 |
| chr9 | 100074958 | C | T | LOC100499484-C9ORF174 | ncRNA_intronic | . | rs117604409 |
| chr9 | 5557708 | T | C | PDCD1LG2 | exonic | . | rs7854413 |
| chr9 | 5557562 | T | C | PDCD1LG2 | intronic | . | rs145952492 |
| chr9 | 5569860 | T | A | PDCD1LG2 | intronic | . | rs7852996 |
| chr9 | 101890980 | A | G | TGFBR1 | intronic | . | rs7041311 |
| chr9 | 101900410 | A | G | TGFBR1 | intronic | . | rs11568778 |
| chr9 | 101908915 | G | A | TGFBR1 | intronic | . | rs334354 |
| chr9 | 101890227 | C | T | TGFBR1 | intronic | . | rs11568753 |

Abbreviation: SNP: single nuclear polymorphism.
