# Supplementary material for: Single Nucleotide Polymorphisms of IL-33 Gene Correlated with Renal Allograft Fibrosis in Kidney Transplant Recipients
Source: J Immunol Res. 2021 Dec 13;2021:8029180. doi: 10.1155/2021/8029180 (PMC8689233; doi:10.1155/2021/8029180)
Supplement: Supplementary 2 — Supplementary Table 2: results of HWE analysis and MAF calculation for included SNPs in our study. [file 8029180.f2.docx]

Supplementary Table 2: Results of HWE analysis and MAF calculation for included SNPs in our study.

| SNPs | MAF | HWE |
| --- | --- | --- |
| rs10975519 | 0.47 | 0.0155 |
| rs1332290 | 0.45 | 0.0463 |
| rs1048274 | 0.27 | 0.7203 |
| rs10975520 | 0.43 | 0.1136 |

Abbreviations: SNP: single nuclear polymorphism; MAF: minor allele frequency; HWE: Hardy Weinberg equilibrium.
