# Supplementary material for: Single Nucleotide Polymorphisms of IL-33 Gene Correlated with Renal Allograft Fibrosis in Kidney Transplant Recipients
Source: J Immunol Res. 2021 Dec 13;2021:8029180. doi: 10.1155/2021/8029180 (PMC8689233; doi:10.1155/2021/8029180)
Supplement: Supplementary 3 — Supplementary Table 3: statistical results of five inheritance models in non-significant tagger SNPs. [file 8029180.f3.docx]

Supplementary Table 3: Statistical results of five inheritance models in non-significant tagger SNPs.

| SNPs | P value | | | | |
| --- | --- | --- | --- | --- | --- |
|  | Codominant model | Dominant model | Recessive model | Overdominant model | log-additive model |
| rs1332290 | 0.250 | 0.099 | 0.698 | 0.238 | 0.211 |
| rs1048274 | 0.252 | 0.831 | 0.155 | 0.154 | 0.510 |
| rs10975520 | 0.087 | 0.290 | 0.161 | 0.031 | 0.946 |

Abbreviation: SNP: single nuclear polymorphisms.
